# Exploring chaos and bifurcation in a discrete prey–predator based on coupled logistic map

**DOI:** 10.1038/s41598-024-62439-8

**Published:** 2024-07-12

**Authors:** Mohammed O. Al-Kaff, Hamdy A. El-Metwally, Abd-Elalim A. Elsadany, Elmetwally M. Elabbasy

**Affiliations:** 1 Department of Mathematics, Faculty of Science, Mansoura University, Mansoura, 35516 Egypt; 2 Basic Science Department, Faculty of Computers and Informatics, Suez Canal University, Ismailia, 41522 Egypt

**Keywords:** Coupled-logistic map, Predator–prey model, Stability, Bifurcation, Marotto’s map, Chaos, Animal behaviour, Dynamical systems, Modularity, Nonlinear dynamics, Numerical simulations

## Abstract

This research paper investigates discrete predator-prey dynamics with two logistic maps. The study extensively examines various aspects of the system’s behavior. Firstly, it thoroughly investigates the existence and stability of fixed points within the system. We explores the emergence of transcritical bifurcations, period-doubling bifurcations, and Neimark-Sacker bifurcations that arise from coexisting positive fixed points. By employing central bifurcation theory and bifurcation theory techniques. Chaotic behavior is analyzed using Marotto’s approach. The OGY feedback control method is implemented to control chaos. Theoretical findings are validated through numerical simulations.

## Introduction

The field of biological mathematics has emphasized the significance of discrete-time models, which provide more relevant mathematical tools for studying dynamical properties compared to continuous models. This relevance is particularly evident when studying populations with non-overlapping generations^1^. Using discrete models is crucial as they can reveal a wider range of dynamic behaviors and enable more efficient computations. The interaction between predators and prey is a crucial aspect of natural ecosystems. Modeling such interactions requires sophisticated approaches that depend on ecosystem conditions and resident populations. The preservation of population balance in these ecosystems has motivated extensive research in this field. The Lotka-Volterra model, developed by Lotka in the United States in 1925^2^ and later expanded by Volterra in Italy in 1926^3^, is a renowned model for prey–predator dynamics. This model describes the behavior of biological systems involving the interaction between two species. Subsequent efforts have been made to further develop and refine this model.

In the 1970s, May’s work shed light on the intriguing discovery that even seemingly basic discrete systems could exhibit complex dynamics^4^. This revelation holds true whether we consider it from a mathematical standpoint or within the context of ecology. The investigation and understanding of complex dynamics within discrete systems have proven to be of immense importance. This principle extends to a diverse range of ecological systems characterized by discreteness, demonstrating the wide applicability and relevance of studying complexity in such systems. By delving into these discrete ecological systems, we gain valuable insights into the intricate dynamics, interactions, and patterns that emerge, thereby deepening our comprehension of the natural world. The logistic map finds valuable applications in both mathematical biology and economics as a growth equation. In mathematical biology, the logistic map is frequently employed to model population dynamics. It captures the interplay between population growth and limited resources, showcasing how populations can reach stable equilibria or exhibit complex oscillations and bifurcations. This allows researchers to study various ecological phenomena, such as predator–prey dynamics, population competition, and the impact of environmental factors on population size. Similarly, in economics, the logistic map serves as a useful tool for modeling economic growth and market dynamics. By considering the population analogy as representing market size or the adoption of a new product, the logistic map can help analyze the saturation of markets, the impact of competition, and the effects of external factors on economic growth. This application enables economists to study the dynamics of market penetration, customer adoption rates, and market saturation levels. Thus, the logistic map serves as a versatile growth equation that provides insights into the dynamics of both biological and economic systems, facilitating a deeper understanding of population behavior, market dynamics, and the complex interplay between growth and limited resources. The study of bifurcations and chaos in discrete prey-predator systems has gained significant attention in recent years due to its relevance in understanding the dynamics of ecological interactions. This literature review aims to provide an overview of the research conducted in this field by examining influential papers that have contributed to our understanding of bifurcations, chaos, and their applications in discrete prey-predator systems. These studies have not only deepened our understanding of the complex dynamics of ecological systems but also have practical implications for population management, conservation biology, and ecological restoration. Conducted a bifurcation analysis of a discrete predator-prey model with a modified Leslie–Gower functional response. They revealed various bifurcation scenarios, including period-doubling bifurcations and chaos in the system dynamics^5^. The influence of parameter variations on the emergence of chaos was examined by researchers in a study on the bifurcation and chaotic dynamics of a discrete predator–prey system with the Beddington–DeAngelis functional response. Their findings indicated that changes in parameters played a role in the occurrence of chaos^6^. The complex behavior of a discrete predator–prey model with the Crowley–Martin functional response was explored, shedding light on the chaotic dynamics within the system^7^. Researchers examined a discrete model of plant–herbivore interaction with a type II Holling response. They investigated the system’s dynamics and performed a qualitative analysis to determine the conditions for stability and bifurcation^8^. Researchers conducted several studies to explore different aspects of discrete predator–prey models. One study focused on a model with a Holling type IV functional response. The researchers analyzed bifurcation behavior and proposed control strategies to suppress chaos^9^. Another study investigated a model with a Holling type III functional response. Bifurcation analysis and chaos control strategies were examined to stabilize the system^10^. In a separate study, researchers analyzed the dynamic behavior of a two-dimensional predator–prey model and suggested control strategies to suppress chaos^11^. Time delay in a predator–prey system was studied in another research. The impact of time delay on system dynamics and bifurcation scenarios leading to chaos were explored^12^. Bifurcation analysis and control strategies were also performed on a coupled logistic model to understand its dynamic behavior and suppress chaos^13^. Another study focused on a prey-predator model with a Holling type-II functional response and prey refuge. Bifurcations and chaos control were investigated to stabilize the system under mutual interference^14^. Lastly, researchers conducted bifurcation analysis and implemented chaos control techniques in a predator-prey system, aiming to understand its behavior and develop effective control methods^15^. These studies contribute to our understanding of predator-prey dynamics and provide insights into controlling chaos in these systems. Discrete prey-predator systems have significant applications in various fields, including population management, conservation biology, ecological restoration, and mathematical modeling. Understanding the dynamics of these systems and the emergence of bifurcations and chaos can provide valuable insights for practical applications. For instance, in population management and conservation biology, studying bifurcations and chaos in prey-predator systems helps identify critical thresholds and tipping points that can lead to population collapses or outbreaks. This knowledge can aid in designing effective strategies for sustainable harvesting, controlling invasive species, and implementing conservation measures to maintain the stability and biodiversity of ecosystems^16,17^. In ecological restoration, discrete prey–predator models and their chaotic dynamics can inform decision-making processes for reintroducing species and restoring ecological balance. By understanding the potential for chaos and its control, restoration efforts can be guided to achieve desired ecological outcomes^18^. Furthermore, mathematical models based on bifurcations and chaos in prey–predator systems serve as valuable tools for predicting and understanding the behavior of real-world ecosystems. These models provide insights into the complex interactions between species, the effects of environmental changes, and the consequences of various management strategies^19–22^. In summary, the applications of discrete prey-predator systems with respect to bifurcations and chaos are wide-ranging and have practical implications in population management, conservation biology, ecological restoration, and mathematical modeling. The knowledge gained from studying these dynamics can inform decision-making processes and contribute to the sustainable management and conservation of ecosystems. These ten papers collectively contribute to our understanding of bifurcations and chaos in discrete prey-predator systems. They provide valuable insights into the complex dynamics of ecological interactions and offer practical applications for managing and conserving populations in real-world ecosystems.

Consider the following discrete-time prey–predator population model based on coupled-logistic map^23,24^:

1 $$\begin{aligned} \left\{ \begin{array}{l} x_{n+1}=ax_{n}(1-x_{n})-cx_{n}y_{n}, \\ y_{n+1}=by_{n}(1-y_{n})+dx_{n}y_{n}, \end{array} \right. \end{aligned}$$

where $x_{n}$ and $y_{n}$ stand for the densities of prey and predator populations, respectively. The term $ax_{n}(1-x_{n})$ represents the rate of increase of the prey populations in the absence of predator. The term $cx_{n}y_{n}$ stands for the rate of decrease due to predation, where the parameter *c* is the predation parameter. The term $by_{n}(1-y_{n})$ represents the rate of decrease in predator populations in the absence of prey. The term $dx_{n}y_{n}$ denotes the growth rate of the predator in the presence of the prey. The coefficients a, b, c and d are positive constants, and $0< x_{n}, y_{n}< 1, 0 < a \le 4$, and $0 < b \le 4$ are the coupling parameters. The term $a\, x_{{n}}$ represents the exponential growth of the prey. The term $a\,x_{n}^{2}$ represents the struggle of preys for food (mating competition between prey males or control of herd leadership). The term $c\,x_{n}y_{n}$ stands for the rate of decrease due to predation. The term $b\,y_{n}$ represents the exponential growth of predator. The term $b\,y_{n}^{2}$ represents the struggle of predators over prey (mating competition between predatory males or control of herd leadership).

It is crucial to highlight that although numerical analysis has been conducted on this system^23,24^, a significant portion of its potential dynamic behaviors, including transcritical bifurcation, period-doubling bifurcation, Neimark–Sacker bifurcation, and others, remain unexplored analytically. In this study, we employ coupled logistic map modeling and difference equations to qualitatively analyze a discrete-time prey–predator model. Our primary objective is to investigate the model’s behavior by exploring diverse parameter values and initial conditions. Specifically, we focus on the emergence of stable equilibria, period doubling, chaotic attractors, as well as the analysis of codimension one bifurcations and chaos control. The results of our analysis reveal a wide range of captivating dynamic behaviors, showcasing the model’s heightened sensitivity to variations in key parameters. These findings hold significant implications for our comprehension of prey–predator systems, underscoring the importance of qualitative analysis in unraveling the complexities inherent in ecological systems. We demonstrate that the model (1) undergoes various types of codimension-one bifurcations through analytical methods. We present bifurcation diagrams and phase portraits to illustrate these bifurcations. Our analysis reveals a multitude of complex and diverse dynamic behaviors, including the presence of limit cycles, periodic solutions, and chaos. These findings highlight the intricate and rich nature of the system’s dynamics.

This paper is structured as follows: “Analysis of fixed points and their stability”  focuses on discussing the stability of the model’s fixed points. In “Local bifurcations analysis”  , we delve into the transcritical bifurcation, period-doubling, and Neimark–Sacker bifurcation, providing a comprehensive analysis of bifurcations at the positive fixed point. “Existence of Marotto’s chaos”  explores the conditions indicating the presence of Marotto’s chaos. The utilization of chaos control strategy to manage the chaotic behavior of the model (1) is presented in “Chaos control” . For clarity, numerical simulations are provided in “Numerical simulations”  to illustrate the key findings. Finally, in “Conclusion” , we summarize our primary discoveries and draw conclusions as we conclude the paper.

## Analysis of fixed points and their stability

In this section, we provide qualitative properties for all fixed points in model (1) as well as the conditions for fixed point asymptotic stability. We have four fixed points, as follows:

(i) When the initial conditions are $p_0(0, 0)$, the total population undergoes extinction.
(ii) In this scenario, the prey becomes extinct, or in the case of the predator only, when $p_1\left( \frac{a - 1}{a}, 0\right)$, where $a>1$.
(iii) When the initial conditions are $p_2(0, \frac{b - 1}{b})$, with $b>1$, the predator becomes extinct, or in the case of the prey only.
(iv) In the case of cohabitation, both the prey and predator coexist when the initial conditions are $p_3\left( \frac{ab - bc - b + c}{ab + cd}, \frac{ab + ad - a - d}{ab + cd}\right)$, subject to the conditions $a>\frac{(c+1)b-c}{b}$, $b>\frac{c}{c+1}$, $b>\frac{a+d-ad}{a}$, and $a(d-1)>d$.

The model (1) is rewritten as follows:

2 $$\begin{aligned} \left\{ \begin{array}{l} x_{n+1}=\eta (x_{n}, y_{n})=ax_{n}(1-x_{n})-cx_{n}y_{n}, \\ y_{n+1}=\mu (x_{n}, y_{n})=by_{n}(1-y_{n})+dx_{n}y_{n}. \end{array} \right. \end{aligned}$$

The Jacobian matrix (J) associated with model (2) at point p(x, y) can be expressed as follows:

3 $$\begin{aligned} J(x,y)=\left( \begin{array}{cc} j_{11} &{} j_{12} \\ j_{21} &{} j_{22} \end{array} \right) , \end{aligned}$$

where

$$\begin{aligned} \begin{array}{ll} j_{11}=\frac{\partial \eta (x_{n}, y_{n})}{\partial x_{n}}|_{(x_{n}, y_{n})}=(1-2x)a - cy, \quad j_{12}=\frac{\partial \eta (x_{n}, y_{n})}{\partial y_{n}}|_{(x_{n}, y_{n})}=-cx, \\ j_{21}=\frac{\partial \mu (x_{n}, y_{n})}{\partial x_{n}}|_{(x_{n}, y_{n})}=dy \quad \text { and } \quad j_{22}=\frac{\partial \mu (x_{n}, y_{n})}{\partial y_{n}}|_{(x_{n}, y_{n})}=(1-2y)b + dx. \end{array} \end{aligned}$$

The auxiliary polynomial is formulated as follows:

4 $$\begin{aligned} {\mathcal{ R}}^{2}+T(x, y){\mathcal{ R}}+D(x, y)=0, \end{aligned}$$

where the quadratic equation (4) has one variable $T(x,y)=-(j_{11}+j_{22})$ and $D(x,y)=j_{11}j_{22}-j_{12}j_{21}$.

### Lemma 1

^25,26^. *Consider the function* ${\mathcal {F}}({\mathcal {R}})={\mathcal {R}}^{2}+T{\mathcal {R}}+D$, *where* ${\mathcal {R}}_1$ *and* ${\mathcal {R}}_2$ *are the two roots of the* *equation* ${\mathcal {F}}({\mathcal {R}})=0$. *Assuming that* ${\mathcal {F}}(1)>0$, *we have the following*:

(i) *The conditions* $\left| {\mathcal {R}}_{1}\right| <1$ *and* $\left| {\mathcal {R}}_{2}\right| <1$ *hold if and only if* ${\mathcal {F}}(-1)>0$ *and* $D<1$.
(ii) *The conditions* $\left| {\mathcal {R}}_{1}\right| <1$ *and* $\left| {\mathcal {R}}_{2}\right| >1$ (*or* $\left| {\mathcal {R}}_{1}\right| >1$ *and* $\left| {\mathcal {R}}_{2}\right| <1$) *hold if and only if* ${\mathcal {F}}(-1)<0$.
(iii) *The conditions* $\left| {\mathcal {R}}_{1}\right| >1$ *and* $\left| {\mathcal {R}}_{2}\right| >1$ *hold if and only if* ${\mathcal {F}}(-1)>0$ *and* $D>1$.
(iv) *The conditions* ${\mathcal {R}}_{1}=-1$ *and* $\left| {\mathcal {R}}_{2}\right| \ne 1$ *hold if and only if* ${\mathcal {F}}(-1)=0$ *and* $T\ne 0,2$.
(v) *The conditions* ${\mathcal {R}}_{1}$ *and* ${\mathcal {R}}_{2}$ *being complex, and* $\left| {\mathcal {R}}_{1}\right| =\left| {\mathcal {R}}_{2}\right| =1$ *hold if and only if* $T^{2}-4D<0$ *and* $D=1$.

### Definition 1

^25,26^. The fixed point *p*(*x*, *y*) is called

1. Sink if $\left| {\mathcal{ R}}_{1}\right| <1$ and $\left| {\mathcal{ R}}_{2}\right| <1$. It is locally asymptotic stable.
2. Saddle if $\left| {\mathcal{ R}}_{1}\right| <1$ and $\left| {\mathcal{ R}}_{2}\right| >1$ (or $\left| {\mathcal{ R}}_{1}\right| >1$ and $\left| {\mathcal{ R}}_{2}\right| <1$). It is locally unstable.
3. Source if $\left| {\mathcal{ R}}_{1}\right| >1$ and $\left| {\mathcal{ R}}_{2}\right| >1$. It is locally unstable.
4. Non-hyperbolic if either $\left| {\mathcal{ R}}_{1}\right| =1$ or $\left| {\mathcal{ R}}_{2}\right| =1$.

By utilizing Lemma 1 and Definition 1, we obtain the following results:

### Theorem 1

*For the simple fixed point* $p_{0}(0, 0)$, *the following statements hold*:

* $P_{0}$ *is a sink if* $0<a<1$ *and* $0<b<1$.
* $P_{0}$ *is a saddle if*
  (1) $$0< a <1$$ *and* $$b>1$$. *Or*
  (2) $$a >1$$ *and* $$0<b<1$$.
* $P_{0}$ *is non-hyperbolic if* $a=1$ *or* $b=1$.
* $P_{0}$ *is a source if* $a>1$ *and* $b>1$.

### Proof

**Figure 1:**
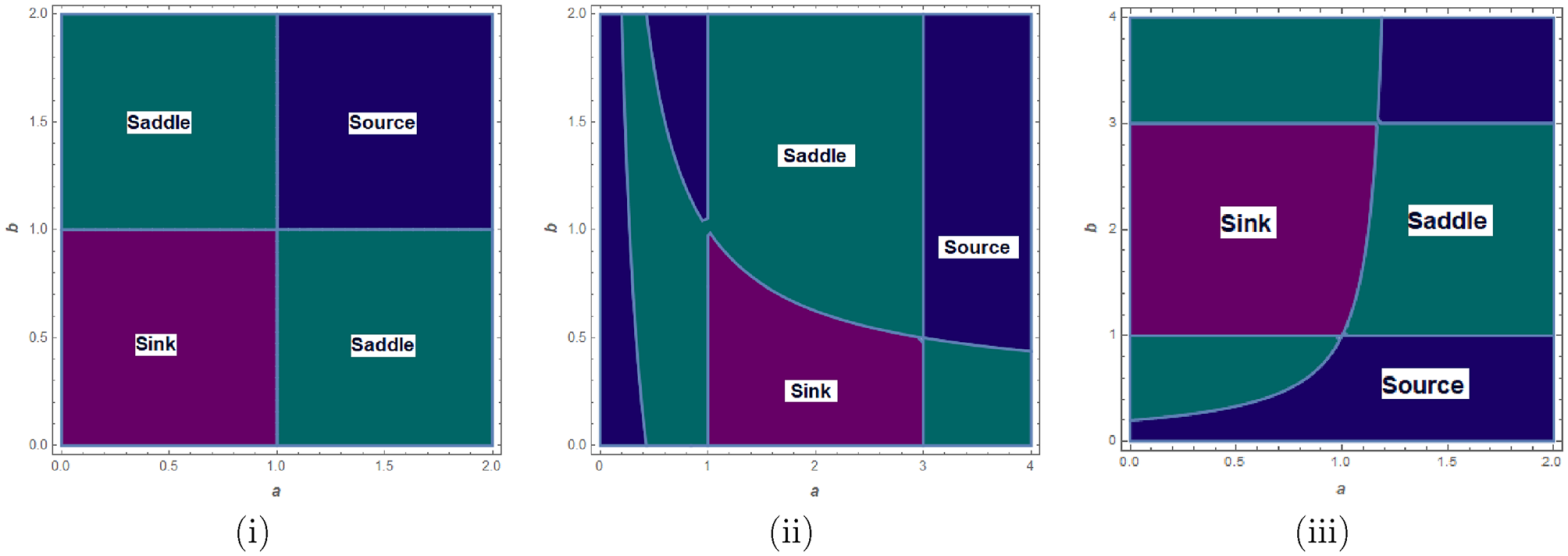
Topological classifications are (i) $p_{0}(0,0)$, (ii) $p_{1}(\dfrac{a - 1}{a}, 0)$, and (iii) $p_{2}(0, \dfrac{b - 1}{b})$.

The Jacobian matrix *J* at $p_{0}(0, 0)$ has the following form:

5 $$\begin{aligned} J(p_{0})=\left( \begin{array}{cc} a &{} 0 \\ 0 &{} b \end{array} \right) , \end{aligned}$$

which have two eigenvalues: ${\mathcal{ R}}_{1}$ $=a$ and ${\mathcal{ R}}_{2}$ $=b$. Obviously, by applying Lemma 1 and Definition 1. Look at Figs. 1(i) and 3. $\square$

### Theorem 2

*The aixel fixed point* $p_{1}\left( \frac{a - 1}{a}, 0\right)$ *exhibits the following characteristics*:

* $P_{1}$ *is a sink if* $1<a<3$ *and* $0<b<min\{\dfrac{d}{a} -1-d,\dfrac{d}{a} +1-d\}$.
* $P_{1}$ *is a saddle if*
  (1) $$1< a <3$$ *and* $$b>max\{\dfrac{d}{a} -1-d,\dfrac{d}{a} +1-d\}$$. *Or*
  (2) $$0< a <1$$ *or* $$3< a <4$$ *and* $$b<min\{\dfrac{d}{a} -1-d,\dfrac{d}{a} +1-d\}$$.
* $P_{1}$ *is non-hyperbolic if* $a=1,3$ *or* $b=\dfrac{d}{a} -1-d, \dfrac{d}{a} +1-d$.
* $P_{1}$ *is a source if*
  (1) $$0< a <1$$ *and* $$b>max\{\dfrac{d}{a} -1-d,\dfrac{d}{a} +1-d\}$$. *Or*
  (2) $$3< a <4$$ *and* $$b>max\{\dfrac{d}{a} -1-d,\dfrac{d}{a} +1-d\}$$.

### Proof

The Jacobian matrix *J* at $p_{1}(\dfrac{a - 1}{a}, 0)$ has the following form:

$$\begin{aligned} J(p_{0})=\left( \begin{array}{cc} 2-a &{} \dfrac{c(1-a)}{a} \\ 0 &{} \dfrac{d(a-1)+ab}{a} \end{array} \right) , \end{aligned}$$

which has two eigenvalues: ${\mathcal{ R}}_{1}$ $=2-a$ and ${\mathcal{ R}}_{2}$ $=\dfrac{ab + ad - d}{a}$. Certainly, by utilizing Lemma 1 and Definition 1, the results can be effectively demonstrated. See in Figure 1(ii) if d = 0.75, and Figure 4. $\square$

### Theorem 3

*For the simple fixed point* $p_{2}(0, \dfrac{b - 1}{b})$, *has the following topological properties*^25,26^:

* $P_{2}$ *is a sink if* $1<b<3$ *and* $0<a<min\{c-\dfrac{b+c}{b},1 + c - \dfrac{c}{b}\}$.
* $P_{2}$ *is a saddle if*
  (1) $$1< b <3$$ *and* $$a>max\{c-\dfrac{b+c}{b},1 + c - \dfrac{c}{b}\}$$. *Or*
  (2) $$0< b <1$$ *or* $$3< b <4$$ *and* $$0<a<min\{c-\dfrac{b+c}{b},1 + c - \dfrac{c}{b}\}$$.
* $P_{2}$ *is non-hyperbolic if* $b=1,3$ *or* $a=c-\dfrac{b+c}{b}, 1 + c - \dfrac{c}{b}$.
* $P_{2}$ *is a source if*
  (1) $$0< b <1$$ *and* $$a>max\{c-\dfrac{b+c}{b},1 + c - \dfrac{c}{b}\}$$. *Or*
  (2) $$3< b <4$$ *and* $$a>max\{c-\dfrac{b+c}{b},1 + c - \dfrac{c}{b}\}$$.

### Proof

The Jacobian matrix *J* evaluated at $p_{2}(0, \frac{b - 1}{b})$ takes the following form:

$$\begin{aligned} J(p_{0})=\left( \begin{array}{cc} \dfrac{c(1-b)+ab}{b} &{} 0 \\ \dfrac{d(b-1)}{b} &{} 2-b \end{array} \right) , \end{aligned}$$

which has two eigenvalues: ${\mathcal{ R}}_{1}$ $=2-b$ and ${\mathcal{ R}}_{2}$ $=\dfrac{ab - bc + c}{{b}}$. Clearly, by employing Lemma 1 and Definition 1, the results can be observed in Fig. 1(iii) if c = 0.25.

The characteristic equation at $P_{3}({\check{x}}, {\check{y}})$ has the form:

$$\begin{aligned} {\mathcal{ R}}^{2}+\mathcal T({\check{x}}, {\check{y}}){\mathcal{ R}}+\mathcal D({\check{x}}, {\check{y}})=0, \end{aligned}$$

where

$$\begin{aligned} \mathcal T({\check{x}}, {\check{y}})= \left( 1-2\,x \right) a-cy+ \left( 1-2\,y \right) b+dx, \end{aligned}$$

and

$$\begin{aligned} D({\check{x}}, {\check{y}})= \left( 2\,x-1 \right) \left( \left( 2\,y-1 \right) b-{ dx} \right) a+{ bcy} \left( 2\,y-1 \right) . \end{aligned}$$

Let

$$\begin{aligned} {\mathcal {F}}({\mathcal{ R}})={\mathcal{ R}}^{2}+\mathcal T({\check{x}}, {\check{y}}){\mathcal{ R}}+\mathcal D({\check{x}}, {\check{y}})=0, \end{aligned}$$

then

$$\begin{aligned} {\mathcal {F}}(1)={\frac{ \left( \left( b+d-1 \right) a-d \right) \left( ab- \left( c+1 \right) b+c \right) }{ab+cd}}, \end{aligned}$$

and

$$\begin{aligned} {\mathcal {F}}(-1)={\frac{b \left( b+d-3 \right) {a}^{2}+ \left( \left( \left( 4-d\right) c-4\,d+9 \right) b+c \left( d-3 \right) -\left( c+3 \right) {b}^{2}\right) a+d \left( \left( c+3 \right) b+3\,c \right) }{ab+cd}}. \end{aligned}$$

$$\square$$

### Theorem 4

*If* $b \left( c+1 \right) <ab+c$ *or* $d+a<a \left( b+d \right)$, *then* $P_{3}({\check{x}}, {\check{y}})$ *has the following topological properties*^25,26^:

* $P_{3}$ *is a sink if*
  (1) $$max\{{\frac{H- \sqrt{{H}^{2}-4\,G}}{2\,b \left( b+d-3 \right) }},{\frac{K+ \sqrt{{K}^{2}-4\,L}}{2\,b \left( b+d-2 \right) }}\}<a<\frac{H+ \sqrt{{H}^{2}-4\,G}}{2\,b \left( b+d-3 \right) }$$. Or
  (2) $${\frac{H- \sqrt{{H}^{2}-4\,G}}{2\,b \left( b+d-3 \right) }}<a<{\frac{K- \sqrt{{K}^{2}-4\,L}}{2\,b \left( b+d-2 \right) }}$$.
* $P_{3}$ *is a saddle if* $a<min\{{\frac{H- \sqrt{{H}^{2}-4\,G}}{2\,b \left( b+d-3 \right) }},{\frac{K+ \sqrt{{K}^{2}-4\,L}}{2\,b \left( b+d-2 \right) }}\}$, *or* $a>\frac{H+ \sqrt{{H}^{2}-4\,G}}{2\,b \left( b+d-3 \right) }$.
* $P_{3}$ *is non-hyperbolic if*
  (1) $$a={\frac{H}{2\,b \left( b+d-3 \right) }}\pm {\frac{ \sqrt{{H}^{2}-4\,G}}{2\,b \left( b+d-3 \right) }}$$. *Or*
  (2) $${K}^{2}>4L$$ *and* $$a={\frac{K \pm \sqrt{{K}^{2}-4\,L}}{2\,b \left( b+d-2 \right) }}$$.
* $P_{3}$ *is a source if*
  (1) $$a < min\{{\frac{H- \sqrt{{H}^{2}-4\,G}}{2\,b \left( b+d-3 \right) }},{\frac{K- \sqrt{{K}^{2}-4\,L}}{2\,b \left( b+d-2 \right) }}\}$$. *Or*
  (2) $$a > max\{{\frac{H+ \sqrt{{H}^{2}-4\,G}}{2\,b \left( b+d-3 \right) }},{\frac{K+ \sqrt{{K}^{2}-4\,L}}{2\,b \left( b+d-2 \right) }}\}$$.

**Figure 2:**
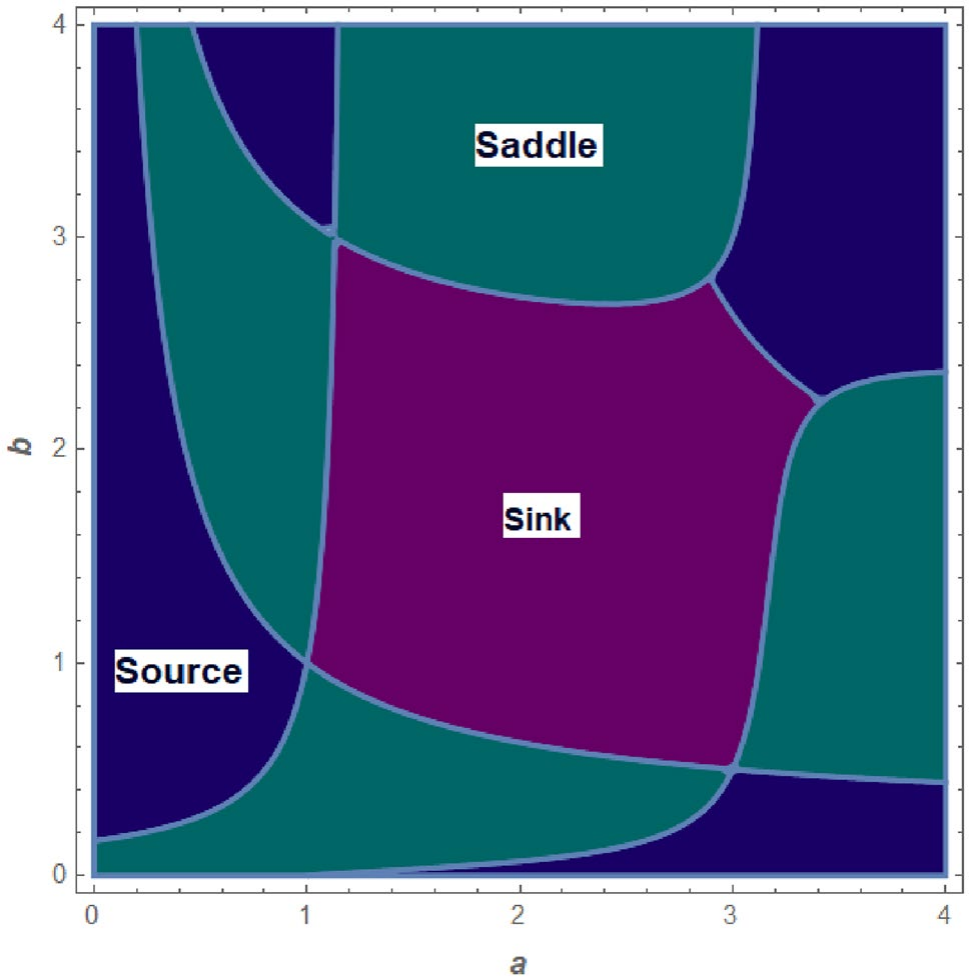
Topological classifications of $P_{3}({\check{x}}, {\check{y}})$ for c = 0.194, d = 0.75.

Where $H= \left( c+3 \right) {b}^{2}+ b\left( \left( d-4 \right) c+4\,d-9 \right) + c\left( 3-d \right)$, $G=bd \left( \left( c+3 \right) b+3\,c \right) \left( b+d-3 \right)$, $K= \left( c+2 \right) {b}^{2}+b \left( c \left( d-3 \right) +3\,d-3 \right) + c\left( 2-d \right)$, $L=bd \left( b+d-2 \right) \left( \left( c+2 \right) b-c \right)$(Figs. 2 and 5).

## Local bifurcations analysis

The purpose of this section is to explore and analyze the different types of bifurcations that occur at the four fixed points, namely $P_{0}$, $P_{1}$, $P_{2}$, and $P_{3}$, in the discrete model (1). To achieve this, we utilize the center manifold theorem and bifurcation theory. For a more detailed exploration of bifurcation theory, readers are encouraged to refer to^27–30^. These bifurcations signify noteworthy changes in the dynamics of the system, providing valuable insights into situations where even minor adjustments in parameters result in significant transformations in predator-prey interactions. Moreover, comprehending the roles of bifurcations aids in the development of efficient conservation and management strategies, ensuring the long-term coexistence of prey and predator populations, and thereby enhancing our understanding of ecosystem dynamics.

### Bifurcation analysis of the fixed point $P_{0}$(0, 0)

The Jacobian matrix evaluated at $P_{0}$(0, 0) is given by:

$$\begin{aligned} J(p_{0})=\left( \begin{array}{cc} a &{} 0 \\ 0 &{} b \end{array} \right) . \end{aligned}$$

If $a = 1$, then $J(P_{0})$ has two eigenvalues: ${\mathcal{ R}}_{1}=1$ and ${\mathcal{ R}}_{2}=b$. If $b\ne 1$, then $\left| {\mathcal{ R}}_{2}\right| \ne 1$ so the conditions of the appearance of transcritical bifurcation at $P_{0}$ is represented by the following Theorem:

#### Theorem 5

*If* $a = 1$, $b\ne 1$, *then the model* (1) *is subject to a transcritical bifurcation at* $P_{0}(0,0)$ *and has only one fixed point*.

#### Proof

By introducing the new dependent variable $\sigma _{n}=a-1$, the model (1) can be transformed into:

6 $$\begin{aligned} \left( \begin{array}{c} x _{n+1} \\ \\ y _{n+1}\\ \\ \sigma _{n+1} \end{array} \right) \rightarrow \left( \begin{array}{ccc} 1 &{} 0 &{} 0 \\ &{} \\ 0 &{} b &{} 0\\ &{} \\ 0 &{} 0 &{} 1 \end{array} \right) \left( \begin{array}{c} x _{n} \\ \\ y _{n}\\ \\ \sigma _{n} \end{array} \right) +\left( \begin{array}{c} {\hat{f}}( x _{n}, y _{n}, \sigma _{n})\\ \\ {\hat{g}}( x _{n}, y _{n}, \sigma _{n}) \\ \\ 0 \end{array} \right) , \end{aligned}$$

where

7 $$\begin{aligned} \begin{array}{l} {\hat{f}}( x _{n}, y _{n}, \sigma _{n})= ( \sigma _{n}- \sigma _{n} x _{n}- x _{n}- c y _{n}) x _{n}, \\ {\hat{g}}( x _{n}, y _{n}, \sigma _{n})=( d x _{n}- b y _{n}) y _{n}. \end{array} \end{aligned}$$

Let

$$\begin{aligned} \begin{array}{l} y _{n} = s( x _{n}, \sigma _{n})={\tilde{\delta }}_{1} x _{n}^{2}+{\tilde{\delta }}_{2} x _{n} \sigma _{n} +{\tilde{\delta }}_{3} \sigma _{n}^{2}+o((\left| x _{n}\right| +\left| \sigma _{n}\right| )^{3}). \end{array} \end{aligned}$$

We restricted the system (6) to center manifold $s( x _{n}, \sigma _{n})$ under the condition

$$\begin{aligned} N\left( s( x _{n}, \sigma _{n})\right) =s( x _{n}+f( x _{n},( x _{n}, \sigma _{n}), \sigma _{n}), \sigma _{n+1})-(b)s( x _{n}, \sigma _{n})-g( x _{n},( x _{n} , \sigma _{n}), \sigma _{n}), \sigma _{n+1})=0. \end{aligned}$$

Thus, we can get the coefficients of the center manifold as follows:

8 $$\begin{aligned} {\tilde{\delta }}_{1}=0, {\tilde{\delta }}_{2}=0 \ and \ {\tilde{\delta }}_{3}=0. \end{aligned}$$

Thus, the center manifold takes the following form:

9 $$\begin{aligned} \begin{array}{l} y _{n} = s( x _{n}, \sigma _{n})={\tilde{\delta }}_{1} x _{n}^{3}+{\tilde{\delta }}_{2} x _{n}^{2} \sigma _{n} +{\tilde{\delta }}_{3} x _{n} \sigma _{n}^{2}+o((\left| x _{n}\right| +\left| \sigma _{n}\right| )^{3}). \end{array} \end{aligned}$$

The map is restricted to the central manifold, which is defined by the following expression:

10 $$\begin{aligned} \begin{array}{l} {\hat{f}}_{1}= x _{n}- x _{n}^{2}+ \sigma _{n} x _{n}- \sigma _{n} x _{n}^{2}- c ({\tilde{\delta }}_{1} x _{n}^{3}+{\tilde{\delta }}_{2} x _{n}^{2} \sigma _{n} +{\tilde{\delta }}_{3} x _{n} \sigma _{n}^{2}) x _{n}+o((\left| x _{n}\right| +\left| \sigma _{n}\right| )^{3}). \end{array} \end{aligned}$$

Here, we define non-zero real numbers as follows:

$$\begin{aligned} \frac{\partial {\hat{f}}_{1}}{\partial x _{n}}=1 \ne 0, \frac{\partial ^{2}{\hat{f}}_{1}}{\partial x _{n}^{2}}=-2 \ne 0 \quad and \quad \frac{\partial ^{2}{\hat{f}}_{1}}{\partial x _{n}\partial \sigma _{n}}=1 \ne 0. \end{aligned}$$

The model (1) undergoes a transcritical bifurcation at $P_{0}(0, 0)$. This completes the proof. $\square$

#### Theorem 6

*If* $a =-1$, $b\ne 1$, *then the model* (1) *is subject to a period-doubling bifurcation at* $P_{0}(0,0)$ *and has only one fixed point*.

#### Proof

If we introduce the new dependent variable $\sigma _{n}=a+1$, the model (1) can be expressed as follows:

11 $$\begin{aligned} \left( \begin{array}{c} x _{n+1} \\ \\ y _{n+1}\\ \\ \sigma _{n+1} \end{array} \right) \rightarrow \left( \begin{array}{ccc} -1 &{} 0 &{} 0 \\ &{} \\ 0 &{} b &{} 0\\ &{} \\ 0 &{} 0 &{} -1 \end{array} \right) \left( \begin{array}{c} x _{n} \\ \\ y _{n}\\ \\ \sigma _{n} \end{array} \right) +\left( \begin{array}{c} {\hat{f}}( x _{n}, y _{n}, \sigma _{n})\\ \\ {\hat{g}}( x _{n}, y _{n}, \sigma _{n}) \\ \\ 0 \end{array} \right) , \end{aligned}$$

where

12 $$\begin{aligned} \begin{array}{l} {\hat{f}}( x _{n}, y _{n}, \sigma _{n})= ( \sigma _{n}- \sigma _{n} x _{n}+ x _{n}- c y _{n}) x _{n}, \\ {\hat{g}}( x _{n}, y _{n}, \sigma _{n})=( d x _{n}- b y _{n}) y _{n}. \end{array} \end{aligned}$$

Let

$$\begin{aligned} \begin{array}{l} y _{n} = s( x _{n}, \sigma _{n})={\tilde{\delta }}_{4} x _{n}^{2}+{\tilde{\delta }}_{5} x _{n} \sigma _{n} +{\tilde{\delta }}_{6} \sigma _{n}^{2}+o((\left| x _{n}\right| +\left| \sigma _{n}\right| )^{3}). \end{array} \end{aligned}$$

We restricted the system (11) to center manifold $s( x _{n}, \sigma _{n})$ under the condition

$$\begin{aligned} N\left( s( x _{n}, \sigma _{n})\right) =s( x _{n}+f( x _{n},( x _{n}, \sigma _{n}), \sigma _{n}), \sigma _{n+1})-(b)s( x _{n}, \sigma _{n})-g( x _{n},( x _{n} , \sigma _{n}), \sigma _{n}), \sigma _{n+1})=0. \end{aligned}$$

Thus, we can get the coefficients of the center manifold as follows:

13 $$\begin{aligned} {\tilde{\delta }}_{4}=0, {\tilde{\delta }}_{5}=0 \ and \ {\tilde{\delta }}_{6}=0. \end{aligned}$$

Therefore, the center manifold can be characterized by the following expression:

14 $$\begin{aligned} \begin{array}{l} y _{n} = s( x _{n}, \sigma _{n})={\tilde{\delta }}_{4} x _{n}^{3}+{\tilde{\delta }}_{5} x _{n}^{2} \sigma _{n} +{\tilde{\delta }}_{6} x _{n} \sigma _{n}^{2}+o((\left| x _{n}\right| +\left| \sigma _{n}\right| )^{3}). \end{array} \end{aligned}$$

The dynamics of the map are confined to the central manifold, which is described by the following expression:

15 $$\begin{aligned} \begin{array}{l} {\hat{f}}_{2}= - x _{n}+ x _{n}^{2}+ \sigma _{n} x _{n}- \sigma _{n} x _{n}^{2}- c ({\tilde{\delta }}_{4} x _{n}^{3}+{\tilde{\delta }}_{5} x _{n}^{2} \sigma _{n} +{\tilde{\delta }}_{6} x _{n} \sigma _{n}^{2}) x _{n}+o((\left| x _{n}\right| +\left| \sigma _{n}\right| )^{3}). \end{array} \end{aligned}$$

Since

$$\begin{aligned} \Psi _{1}=\left( 2\frac{\partial ^{2}{\hat{f}}_{2}}{\partial x _{n}\partial \sigma _{n}}+ \frac{\partial {\hat{f}}_{2}}{\partial \sigma _{n}}\frac{\partial ^{2}{\hat{f}}_{2}}{ \partial x _{n}^{2}}\right) |_{\left( 0,0\right) }=2\ne 0, \end{aligned}$$

and

$$\begin{aligned} \Psi _{2}=\left( \frac{1}{3}\frac{\partial ^{3}{\hat{f}}_{2}}{\partial x _{n}^{3}}+ \frac{1}{2}\left( \frac{\partial ^{2}{\hat{f}}_{2}}{\partial x _{n}^{2}}\right) ^{2}\right) |_{\left( 0,0\right) }=2\ne 0. \end{aligned}$$

The model (1) undergoes a period-doubling bifurcation at $P_{0}(0, 0)$. This completes the proof. $\square$

### Bifurcation analysis of the fixed point $P_{1}\left( \frac{a - 1}{a}, 0\right)$

The Jacobian matrix at $P_{1}(\dfrac{a - 1}{a}, 0)$ reads

$$\begin{aligned} J(p_{1})=\left( \begin{array}{cc} 2-a &{} \dfrac{c(1-a)}{a} \\ 0 &{} \dfrac{d(a - 1)+ab}{a} \end{array} \right) . \end{aligned}$$

If $b =\dfrac{a+d-ad}{a}$, then $J(P_{1})$ has two eigenvalues: ${\mathcal{ R}}_{1}=1$ and ${\mathcal{ R}}_{2}=2-a$. If $a\ne 1,3$, then $\left| {\mathcal{ R}}_{2}\right| \ne 1$ so the conditions of the appearance of transcritical bifurcation at $P_{1}$ is summarized by the following theorem:

#### Theorem 7

*If* $b =\dfrac{a+d-ad}{a}$, $a\ne 1,3$, *then the model* (1) *is subject to a transcritical bifurcation at* $P_{1}(\dfrac{a - 1}{a}, 0)$ *and has only one fixed point*.

#### Proof

By introducing the new dependent variables $\sigma _{n}=b-\frac{a+d-ad}{a}$, $\alpha _{n} = x_{n}-\frac{a - 1}{a}$, and $\beta _{n} = y_{n}$, the model (1) can be transformed into:

16 $$\begin{aligned} \left( \begin{array}{l} \alpha _{n+1} \\ \\ \beta _{n+1}\\ \\ \sigma _{n+1} \end{array} \right) \rightarrow \left( \begin{array}{ccc} 2-a &{} \dfrac{c(1-a)}{a} &{} 0 \\ &{} \\ 0 &{} 1 &{} 0\\ &{} \\ 0 &{} 0 &{} 1 \end{array} \right) \left( \begin{array}{c} \alpha _{n} \\ \\ \beta _{n}\\ \\ \sigma _{n} \end{array} \right) +\left( \begin{array}{c} -(a \alpha _{n}+c \beta _{n}) \alpha _{n}\\ \\ ( \sigma _{n} \alpha _{n}+\dfrac{(a-1)^2 \sigma _{n}+a^2(1-b) \alpha _{n}}{a(a-1)}-b \beta _{n}) \beta _{n}\\ \\ 0 \end{array} \right) . \end{aligned}$$

Let

$$\begin{aligned} T=\left( \begin{array}{ccc} 1 &{} c &{} 0 \\ 0 &{} -a &{} 0\\ 0 &{} 0 &{} 1\\ \end{array} \right) , \end{aligned}$$

and use the transformation

17 $$\begin{aligned} \left( \begin{array}{c} \alpha _{n} \\ \beta _{n}\\ \sigma _{n} \end{array} \right) = \begin{array}{ccc} T \end{array} \left( \begin{array}{c} u _{n} \\ v _{n}\\ \xi _{n} \end{array} \right) , \end{aligned}$$

system (16) becomes

18 $$\begin{aligned} \left( \begin{array}{c} u _{n+1} \\ \\ v _{n+1}\\ \\ \xi _{n+1} \end{array} \right) \rightarrow \left( \begin{array}{ccc} 2-a &{} 0 &{} 0 \\ &{} \\ 0 &{} 1 &{} 0\\ &{} \\ 0 &{} 0 &{} 1 \end{array} \right) \left( \begin{array}{c} u _{n} \\ \\ v _{n}\\ \\ \xi _{n} \end{array} \right) +\left( \begin{array}{c} {\hat{f}}( u _{n}, v _{n}, \xi _{n})\\ \\ {\hat{g}}( u _{n}, v _{n}, \xi _{n}) \\ \\ 0 \end{array} \right) , \end{aligned}$$

where

19 $$\begin{aligned} \begin{array}{l} {\hat{f}}( x _{n}, y _{n}, \xi _{n})= -a u _{n}^2 -\dfrac{ac(a-b) u _{n} v _{n}}{a-1}-c \xi _{n} u _{n} v _{n}-c^2 \xi _{n} v _{n}^2-\dfrac{ac(ab-(c+1)b+c) v _{n}^2}{a-1} \\ \qquad \qquad \quad \quad \; -\dfrac{c(a-1) \xi _{n} v _{n}}{a} , \\ {\hat{g}}( x _{n}, y _{n}, \xi _{n})=\dfrac{a(1-b) u _{n} v _{n}}{a-1}+ \xi _{n} u _{n} v _{n}+c \xi _{n} v _{n}^2+\dfrac{a(ab-(c+1)b+c) v _{n}^2}{a-1} \\ \qquad \qquad \quad \quad \; +\dfrac{(a-1) \xi _{n} v _{n}}{a}. \end{array} \end{aligned}$$

Consider

$$\begin{aligned} \begin{array}{l} u _{n} = s( v _{n}, \xi _{n})={\hat{\delta }}_{7} v _{n}^{2}+{\hat{\delta }}_{8} v _{n} \xi _{n} +{\hat{\delta }}_{9} \xi _{n}^{2}+o((\left| v _{n}\right| +\left| \xi _{n}\right| )^{3}). \end{array} \end{aligned}$$

It must satisfy

$$\begin{aligned} s( v _{n}+g(( v _{n}, \xi _{n}), v _{n}, \xi _{n}), \xi _{n+1})=(2-a)s( v _{n}, \xi _{n})+f(( v _{n}, \xi _{n}), v _{n}, \xi _{n}), \xi _{n+1}). \end{aligned}$$

Thus, we can get that

20 $$\begin{aligned} {\hat{\delta }}_{7}=-\dfrac{ac((a-c-1)b+c)}{(a-1)^2}, {\hat{\delta }}_{8}=-\dfrac{c}{a} \quad and \quad {\hat{\delta }}_{9}=0. \end{aligned}$$

And the map is limited to the central manifold that was given by

21 $$\begin{aligned} \begin{array}{l} {\hat{f}}_{3}= v _{n}+\dfrac{a(ab-(c+1)b+c) v _{n}^2}{a-1}+\dfrac{(a-1) \xi _{n} v _{n}}{a}+o((\left| v _{n}\right| +\left| \xi _{n}\right| )^{3}). \end{array} \end{aligned}$$

Since

$$\begin{aligned} \frac{\partial {\hat{f}}_{3}}{\partial v _{n}}=1 \ne 0, \frac{\partial ^{2}{\hat{f}}_{3}}{\partial v _{n}^{2}}=\dfrac{2a(ab-(c+1)b+c)}{a-1} \ne 0 \quad and \quad \frac{\partial ^{2}{\hat{f}}_{3}}{\partial v _{n}\partial \xi _{n}}=\dfrac{a-1}{a} \ne 0. \end{aligned}$$

The model (1) undergoes a transcritical bifurcation at $P_{1}(\dfrac{a - 1}{a}, 0)$. This completes the proof.$\square$

#### Theorem 8

*If* $b =\dfrac{d-a-ad}{a}$, $a\ne 1,3$, *then the model* (1) *is subject to a period-doubling bifurcation at* $P_{1}(\dfrac{a - 1}{a}, 0)$ *and has only one fixed point*.

#### Proof

Let parameter $\sigma _{n}=b-\dfrac{d-a-ad}{a}$, $\alpha _{n} = x _{n}-\dfrac{a - 1}{a}$   and   $\beta _{n} = y _{n}$ be a new dependent variable. Then, the model (1) becomes

22 $$\begin{aligned} \left( \begin{array}{l} \alpha _{n+1} \\ \\ \beta _{n+1}\\ \\ \sigma _{n+1} \end{array} \right) \rightarrow \left( \begin{array}{ccc} 2-a &{} \dfrac{c(1-a)}{a} &{} 0 \\ &{} \\ 0 &{} -1 &{} 0\\ &{} \\ 0 &{} 0 &{} -1 \end{array} \right) \left( \begin{array}{c} \alpha _{n} \\ \\ \beta _{n}\\ \\ \sigma _{n} \end{array} \right) +\left( \begin{array}{c} -(a \alpha _{n}+c \beta _{n}) \alpha _{n}\\ \\ ( \sigma _{n} \alpha _{n}-\dfrac{a^2(1+b) \alpha _{n}-(a-1)^2 \sigma _{n}}{a(a-1)}-b \beta _{n}) \beta _{n}\\ \\ 0 \end{array} \right) . \end{aligned}$$

Let

$$\begin{aligned} T=\left( \begin{array}{ccc} 1 &{} c(a-1) &{} 0 \\ 0 &{} a(3-a) &{} 0\\ 0 &{} 0 &{} 1\\ \end{array} \right) , \end{aligned}$$

and use the transformation

23 $$\begin{aligned} \left( \begin{array}{c} \alpha _{n} \\ \beta _{n}\\ \sigma _{n} \end{array} \right) = \begin{array}{ccc} T \end{array} \left( \begin{array}{c} u _{n} \\ v _{n}\\ \xi _{n} \end{array} \right) , \end{aligned}$$

system (22) becomes

24 $$\begin{aligned} \left( \begin{array}{c} u _{n+1} \\ \\ v _{n+1}\\ \\ \xi _{n+1} \end{array} \right) \rightarrow \left( \begin{array}{ccc} 2-a &{} 0 &{} 0 \\ &{} \\ 0 &{} -1 &{} 0\\ &{} \\ 0 &{} 0 &{} -1 \end{array} \right) \left( \begin{array}{c} u _{n} \\ \\ v _{n}\\ \\ \xi _{n} \end{array} \right) +\left( \begin{array}{c} {\hat{f}}( u _{n}, v _{n}, \xi _{n})\\ \\ {\hat{g}}( u _{n}, v _{n}, \xi _{n}) \\ \\ 0 \end{array} \right) , \end{aligned}$$

where

25 $$\begin{aligned} \begin{array}{l} {\hat{f}}( x _{n}, y _{n}, \xi _{n})= -a u _{n}^2 -ac(a-b) u _{n} v _{n}-c(a-1) \xi _{n} u _{n} v _{n}-c^2(a-1)^2 \xi _{n} v _{n}^2 \\ \qquad \qquad \quad \quad \; -ac(a-1)(ab-(c+3)b+c) v _{n}^2-\dfrac{c(a-1)^2 \xi _{n} v _{n}}{a} , \\ {\hat{g}}( x _{n}, y _{n}, \xi _{n})=a((a-c-3)b-c) v _{n}^2-\dfrac{a(b+1) u _{n} v _{n}}{a-1}+ \xi _{n} u _{n} v _{n}+c(a-1) \xi _{n} v _{n}^2 \\ \qquad \qquad \quad \quad \; +\dfrac{(a-1) \xi _{n} v _{n}}{a}. \end{array} \end{aligned}$$

Consider

$$\begin{aligned} \begin{array}{l} u _{n} = s( v _{n}, \xi _{n})={\hat{\delta }}_{10} v _{n}^{2}+{\hat{\delta }}_{11} v _{n} \xi _{n} +{\hat{\delta }}_{12} \xi _{n}^{2}+o((\left| v _{n}\right| +\left| \xi _{n}\right| )^{3}). \end{array} \end{aligned}$$

It must satisfy

$$\begin{aligned} s( v _{n}+g(( v _{n}, \xi _{n}), v _{n}, \xi _{n}), \xi _{n+1})=(2-a)s( v _{n}, \xi _{n})+f(( v _{n}, \xi _{n}), v _{n}, \xi _{n}), \xi _{n+1}). \end{aligned}$$

Thus, we can get that

26 $$\begin{aligned} {\hat{\delta }}_{10}=-((a-c-3)b+c)ac, {\hat{\delta }}_{11}=-\dfrac{c(a-1)^2}{a(a-3)} \quad and \quad {\hat{\delta }}_{12}=0. \end{aligned}$$

And the map is limited to the central manifold that was given by

27 $$\begin{aligned} \begin{array}{l} {\hat{f}}_{4}= - v _{n}+\dfrac{(a-1) \xi _{n} v _{n}}{a}+a((a-c-3)b-c) v _{n}^2- {\dfrac{6 c {a}^{2} \left( b+1 \right) \left( \left( a-c-3 \right) b+ c \right) }{a-1}}v_{n}^3 +\, o((\left| v _{n}\right| +\left| \xi _{n}\right| )^{4}). \end{array} \end{aligned}$$

Since

$$\begin{aligned} \Psi _{1}=\left( 2\frac{\partial ^{2}{\hat{f}}_{4}}{\partial v _{n}\partial \xi _{n}}+ \frac{\partial {\hat{f}}_{4}}{\partial \xi _{n}}\frac{\partial ^{2}{\hat{f}}_{4}}{ \partial v _{n}^{2}}\right) |_{\left( 0,0\right) }=2-\dfrac{2}{a}\ne 0, \end{aligned}$$

and

$$\begin{aligned} \Psi _{2}=\left( \frac{1}{3}\frac{\partial ^{3}{\hat{f}}_{4}}{\partial v _{n}^{3}}+ \frac{1}{2}(\frac{\partial ^{2}{\hat{f}}_{4}}{\partial v _{n}^{2}})^{2}\right) |_{\left( 0,0\right) }=2a^2((a-c-3)b-c)^2+\dfrac{ 2ca^2(b+1)((a-c-3)b+c)}{a-1}\ne 0. \end{aligned}$$

The model (1) undergoes a period-doubling bifurcation at $P_{1}(\dfrac{a - 1}{a}, 0)$. This completes the proof. $\square$

### Bifurcation analysis of the fixed point $P_{2}\left( 0, \frac{b - 1}{b}\right)$

The Jacobian matrix at $P_{2}(0, \dfrac{b - 1}{b})$ reads

$$\begin{aligned} J(p_{2})=\left( \begin{array}{cc} \dfrac{c(1-b)+ab}{b} &{} 0 \\ \dfrac{d(b-1)}{b} &{} 2-b \end{array} \right) , \end{aligned}$$

If $a =\dfrac{bc+b-c}{b}$, then $J(P_{2})$ has two eigenvalues: ${\mathcal{ R}}_{1}=1$ and ${\mathcal{ R}}_{2}=2-a$. If $b\ne 1,3$, then $\left| {\mathcal{ R}}_{2}\right| \ne 1$ so the conditions of the appearance of transcritical bifurcation at $P_{2}$ is represented by the following theorem.

#### Theorem 9

*If* $a =\dfrac{bc+b-c}{b}$, $a\ne 1,3$, *then the model* (1) *is subject to a transcritical bifurcation at* $P_{2}(0, \dfrac{b - 1}{b})$ *and has only one fixed point*.

#### Proof

Let parameter $\sigma _{n}=a-\dfrac{bc+b-c}{b}$, $\alpha _{n} = x _{n}$   and   $\beta _{n} = y _{n}-\dfrac{b - 1}{b}$ be a new dependent variable. Then, the model (1) becomes

28 $$\begin{aligned} \left( \begin{array}{l} \alpha _{n+1} \\ \\ \beta _{n+1}\\ \\ \sigma _{n+1} \end{array} \right) \rightarrow \left( \begin{array}{ccc} 1 &{} 0 &{} 0 \\ &{} \\ \dfrac{d(b-1)}{b} &{} 2-b &{} 0\\ &{} \\ 0 &{} 0 &{} 1 \end{array} \right) \left( \begin{array}{c} \alpha _{n} \\ \\ \beta _{n}\\ \\ \sigma _{n} \end{array} \right) +\left( \begin{array}{c} (- \sigma _{n} \beta _{n}-\dfrac{(b-1)^2 \sigma _{n}-b^2(a-1) \beta _{n}}{b(b-1)}-a \alpha _{n}) \alpha _{n}\\ \\ (d \alpha _{n}-b \beta _{n}) \beta _{n}\\ \\ 0 \end{array} \right) . \end{aligned}$$

Let

$$\begin{aligned} T=\left( \begin{array}{ccc} b &{} 0 &{} 0 \\ d &{} 1 &{} 0\\ 0 &{} 0 &{} 1\\ \end{array} \right) , \end{aligned}$$

and use the transformation

29 $$\begin{aligned} \left( \begin{array}{c} \alpha _{n} \\ \beta _{n}\\ \sigma _{n} \end{array} \right) = \begin{array}{ccc} T \end{array} \left( \begin{array}{c} u _{n} \\ v _{n}\\ \xi _{n} \end{array} \right) , \end{aligned}$$

system (28) becomes

30 $$\begin{aligned} \left( \begin{array}{c} u _{n+1} \\ \\ v _{n+1}\\ \\ \xi _{n+1} \end{array} \right) \rightarrow \left( \begin{array}{ccc} 1 &{} 0 &{} 0 \\ &{} \\ 0 &{} 2-b &{} 0\\ &{} \\ 0 &{} 0 &{} 1 \end{array} \right) \left( \begin{array}{c} u _{n} \\ \\ v _{n}\\ \\ \xi _{n} \end{array} \right) +\left( \begin{array}{c} {\hat{f}}( u _{n}, v _{n}, \xi _{n})\\ \\ {\hat{g}}( u _{n}, v _{n}, \xi _{n}) \\ \\ 0 \end{array} \right) , \end{aligned}$$

where

31 $$\begin{aligned} \begin{array}{l} {\hat{f}}( x _{n}, y _{n}, \xi _{n})=-\dfrac{b((b+d-1)a-d) u _{n}^2}{b-1}-\dfrac{b(a-1) u _{n} v _{n}}{b-1}-\dfrac{(b-1) \xi _{n} u _{n}}{b}- \xi _{n} u _{n} v _{n} \\ \qquad \qquad \quad \quad \; -d u _{n}^2 \xi _{n}, \\ {\hat{g}}( x _{n}, y _{n}, \xi _{n})=\dfrac{bd((b+d-1)a-d) u _{n}^2}{b-1}+\dfrac{bd(a-b) u _{n} v _{n}}{b-1}+\dfrac{d(b-1) \xi _{n} u _{n}}{b}+d \xi _{n} u _{n} v _{n} \\ \qquad \qquad \quad \quad \; -b v _{n}^2+d^2 u _{n}^2 \xi _{n}. \end{array} \end{aligned}$$

Consider

$$\begin{aligned} \begin{array}{l} v _{n} = s( u _{n}, \xi _{n})={\hat{\delta }}_{13} u _{n}^{2}+{\hat{\delta }}_{14} u _{n} \xi _{n} +{\hat{\delta }}_{15} \xi _{n}^{2}+o((\left| u _{n}\right| +\left| \xi _{n}\right| )^{3}). \end{array} \end{aligned}$$

It must satisfy

$$\begin{aligned} s( u _{n}+f( u _{n},( u _{n}, \xi _{n}), \xi _{n}), \xi _{n+1})=(2-b)s( u _{n}, \xi _{n})+g( u _{n},( u _{n}, \xi _{n}), \xi _{n}), \xi _{n+1}). \end{aligned}$$

Thus, we can get that

32 $$\begin{aligned} {\hat{\delta }}_{13}={\frac{bd \left( \left( b+d-1 \right) a-d \right) }{ \left( b-1\right) ^{2}}}, {\hat{\delta }}_{14}=\dfrac{d}{b} \quad and \quad {\hat{\delta }}_{15}=0. \end{aligned}$$

And the map is limited to the central manifold that was given by

33 $$\begin{aligned} \begin{array}{l} {\hat{f}}_{5}= u _{n}+\dfrac{b((b+d-1)a-d) u _{n}^2}{(b-1)}-\dfrac{(b-1) \xi _{n} u _{n}}{b}+o((\left| v _{n}\right| +\left| \xi _{n}\right| )^{3}). \end{array} \end{aligned}$$

Since

$$\begin{aligned} \frac{\partial {\hat{f}}_{5}}{\partial u _{n}}=1 \ne 0, \frac{\partial ^{2}{\hat{f}}_{5}}{\partial u _{n}^{2}}=-\dfrac{2b((b+d-1)a-d)}{b-1} \ne 0 \quad and \quad \frac{\partial ^{2}{\hat{f}}_{5}}{\partial u _{n}\partial \xi _{n}}=-\dfrac{(b-1)}{b} \ne 0. \end{aligned}$$

The model (1) undergoes a transcritical bifurcation at $P_{2}(0, \dfrac{b - 1}{b})$. This completes the proof. $\square$

#### Theorem 10

*If* $a =\dfrac{bc-b-c}{b}$, $a\ne 1,3$, *then the model* (1) *is subject to a period-doubling bifurcation at* $P_{2}(0, \dfrac{b - 1}{b})$ *and has only one fixed point*.

#### Proof

By introducing the new dependent variables $\sigma _{n}=a-\frac{bc-b-c}{b}$, $\alpha _{n} = x_{n}$, and $\beta _{n} = y_{n}-\frac{b - 1}{b}$, the model (1) can be transformed into:

34 $$\begin{aligned} \left( \begin{array}{l} \alpha _{n+1} \\ \\ \beta _{n+1}\\ \\ \sigma _{n+1} \end{array} \right) \rightarrow \left( \begin{array}{ccc} -1 &{} 0 &{} 0 \\ &{} \\ \dfrac{d(b-1)}{b} &{} 2-b &{} 0\\ &{} \\ 0 &{} 0 &{} -1 \end{array} \right) \left( \begin{array}{c} \alpha _{n} \\ \\ \beta _{n}\\ \\ \sigma _{n} \end{array} \right) +\left( \begin{array}{c} ( \sigma _{n} \beta _{n}-\dfrac{(b-1)^2 \sigma _{n}-b^2(a+1) \beta _{n}}{b(b-1)}-a \alpha _{n}) \alpha _{n}\\ \\ (d \alpha _{n}-b \beta _{n}) \beta _{n}\\ \\ 0 \end{array} \right) . \end{aligned}$$

Let

$$\begin{aligned} T=\left( \begin{array}{ccc} b(b-3) &{} 0 &{} 0 \\ d(b-1) &{} 1 &{} 0\\ 0 &{} 0 &{} 1\\ \end{array} \right) , \end{aligned}$$

and use the transformation

35 $$\begin{aligned} \left( \begin{array}{c} \alpha _{n} \\ \beta _{n}\\ \sigma _{n} \end{array} \right) = \begin{array}{ccc} T \end{array} \left( \begin{array}{c} u _{n} \\ v _{n}\\ \xi _{n} \end{array} \right) , \end{aligned}$$

system (34) becomes

36 $$\begin{aligned} \left( \begin{array}{c} u _{n+1} \\ \\ v _{n+1}\\ \\ \xi _{n+1} \end{array} \right) \rightarrow \left( \begin{array}{ccc} -1 &{} 0 &{} 0 \\ &{} \\ 0 &{} 2-b &{} 0\\ &{} \\ 0 &{} 0 &{} -1 \end{array} \right) \left( \begin{array}{c} u _{n} \\ \\ v _{n}\\ \\ \xi _{n} \end{array} \right) +\left( \begin{array}{c} {\hat{f}}( u _{n}, v _{n}, \xi _{n})\\ \\ {\hat{g}}( u _{n}, v _{n}, \xi _{n}) \\ \\ 0 \end{array} \right) , \end{aligned}$$

where

37 $$\begin{aligned} \begin{array}{l} {\hat{f}}( x _{n}, y _{n}, \xi _{n})=-b((b+d-3)a+d) u _{n}^2-\dfrac{b(a+1) u _{n} v _{n}}{b-1}-\dfrac{(b-1) \xi _{n} u _{n}}{b}- \xi _{n} u _{n} v _{n} \\ \qquad \qquad \quad \quad \; -d(b-1) u _{n}^2 \xi _{n}, \\ {\hat{g}}( x _{n}, y _{n}, \xi _{n})=b d \left( b-1 \right) \left( \left( b+d-3 \right) a-d \right) {u_{{n}}}^{2}+ bd\left( a-b \right) u _{n} v _{n}+\dfrac{d(b-1)^2 \xi _{n} u _{n}}{b} \\ \qquad \qquad \quad \quad \;+ \;d \left( b-1 \right) \xi _{n} u _{n} v _{n} -b v _{n}^2+d^2(b-1)^2 u _{n}^2 \xi _{n}. \end{array} \end{aligned}$$

Consider

$$\begin{aligned} \begin{array}{l} v _{n} = s( u _{n}, \xi _{n})={\hat{\delta }}_{16} u _{n}^{2}+{\hat{\delta }}_{17} u _{n} \xi _{n} +{\hat{\delta }}_{18} \xi _{n}^{2}+o((\left| u _{n}\right| +\left| \xi _{n}\right| )^{3}). \end{array} \end{aligned}$$

It must satisfy

$$\begin{aligned} s(- u _{n}+f( u _{n},( u _{n}, \xi _{n}), \xi _{n}), \xi _{n+1})=(2-b)s( u _{n}, \xi _{n})+g( u _{n},( u _{n}, \xi _{n}), \xi _{n}), \xi _{n+1}). \end{aligned}$$

Thus, we can get that

38 $$\begin{aligned} {\hat{\delta }}_{16}={ bd}\, \left( ab+ad-3\,a-d \right) , {\hat{\delta }}_{17}=\dfrac{d(b-1)^2}{b(b-3)} \quad and \quad {\hat{\delta }}_{18}=0. \end{aligned}$$

The map is restricted to the central manifold, which is described by the following expression:

39 $$\begin{aligned} \begin{array}{l} {\hat{f}}_{6}= - u _{n}-\dfrac{(b-1) \xi _{n} u _{n}}{b}-b((b-d-3)a-d) u _{n}^2-{\dfrac{{b}^{2} \left( a+1 \right) \left( ab+ad-3\,a-d \right) d{u}^ {3}}{b-1}} \\ \qquad +\, o((\left| v _{n}\right| +\left| \xi _{n}\right| )^{4}). \end{array} \end{aligned}$$

Since

$$\begin{aligned} \Psi _{1}=\left( 2\frac{\partial ^{2}{\hat{f}}_{6}}{\partial u _{n}\partial \xi _{n}}+ \frac{\partial {\hat{f}}_{6}}{\partial \xi _{n}}\frac{\partial ^{2}{\hat{f}}_{6}}{ \partial u _{n}^{2}}\right) |_{\left( 0,0\right) }=\dfrac{2(1-b)}{b}\ne 0, \end{aligned}$$

and

$$\begin{aligned} \Psi _{2}=\left( \frac{1}{3}\frac{\partial ^{3}{\hat{f}}_{6}}{\partial u _{n}^{3}}+ \frac{1}{2}(\frac{\partial ^{2}{\hat{f}}_{6}}{\partial u _{n}^{2}})^{2}\right) |_{\left( 0,0\right) }=2\,{b}^{2}\left( \left( b+d-3 \right) a+d \right) ^{2} -{\frac{2 d{b}^{2} \left( a+1 \right) \left( \left( b+d-3 \right) a-d \right) }{b-1}}. \end{aligned}$$

The model (1) undergoes a period-doubling bifurcation at $P_{2}(0, \dfrac{b - 1}{b})$. This completes the proof. $\square$

### Bifurcation analysis of $P_{3}({\check{x}}, {\check{y}})$

In this subsection, we investigate the bifurcation analysis centered on $P_{3}({\check{x}}, {\check{y}})$. We consider that

$$\begin{aligned} F_{P_{3}}=\left\{ \begin{array}{c} (a, b, c, d):a=a_{1}=\dfrac{H \pm \sqrt{{H}^{2}-4\,G}}{2\,b \left( b+d-3 \right) },{H}^{2}>4\,G \end{array} \right\} , \end{aligned}$$

and

$$\begin{aligned} N_{P_{3}}=\left\{ \begin{array}{c} (a, b, c, d):a=a_{2}={\dfrac{K \pm \sqrt{{K}^{2}-4\,L}}{2\,b \left( b+d-2 \right) }},4L>{K}^{2} \end{array} \right\} . \end{aligned}$$

To explore the period-doubling bifurcation of model (1) at $P_{3}({\check{x}}, {\check{y}})$ when its parameters change in the neighborhood of $F_{P_{3}}$, we study the model (1) for $(a, b, c, d) \in F_{P_{3}}$ as follows:

40 $$\begin{aligned} \left\{ \begin{array}{l} x_{n+1}=a_{1}x_{n}(1-x_{n})-cx_{n}y_{n}, \\ y_{n+1}=by_{n}(1-y_{n})+dx_{n}y_{n}. \\ \end{array} \right. \end{aligned}$$

The eigenvalues for $P_{3}({\check{x}}, {\check{y}})$ of the model (40) are ${\mathcal{ R}}_{1}=-1$ and $\left| {\mathcal{ R}}_{2}\right| \ne 1$. By selecting $a_{*}$ as the bifurcation parameter and introducing a small perturbation $a_{1}$ to the Eq. (40), we obtain:

41 $$\begin{aligned} \left\{ \begin{array}{l} x_{n+1}=(a_{1}+a_{*})x_{n}(1-x_{n})-cx_{n}y_{n}, \\ y_{n+1}=by_{n}(1-y_{n})+dx_{n}y_{n}, \\ \end{array} \right. \end{aligned}$$

where $$a_{*}\ll 1$$.

Let $u=x-{\check{x}}$, $u=y-{\check{y}}$. Then system (41) converted to

42 $$\begin{aligned} \left( \begin{array}{c} {u} \\ \\ {v} \end{array} \right) {\tiny \rightarrow }\left( \begin{array}{c} {\mathcal {E}}_{11}u+{\mathcal {E}}_{12}v+{\mathcal {E}}_{13}uv+{\mathcal {E}}_{14}u^{2} +{\hat{P}}_{1}ua_{*}+{\hat{P}} _{2}u^{2}a_{*} \\ \\ {\mathcal {E}}_{21}u+{\mathcal {E}}_{22}v+{\mathcal {E}}_{23}uv+{\mathcal {E}}_{24}v^{2} \\ \end{array} \right) , \end{aligned}$$

where

43 $$\begin{aligned} \left\{ \begin{array}{l} {\mathcal {E}}_{11}=a-2a{\check{x}}-c{\check{y}},{\mathcal {E}}_{12}=-c{\check{x}}, {\mathcal {E}} _{13}=-c,{\mathcal {E}} _{14}=-a,{\hat{P}}_{1}=1-2{\check{x}},{\hat{P}}_{2}=-1,\\ {\mathcal {E}} _{21}=d{\check{y}} ,{\mathcal {E}}_{22}=b-2b{\check{y}}+d{\check{x}} ,{\mathcal {E}} _{23}=d ,{\mathcal {E}}_{24}=-b. \end{array} \right. \end{aligned}$$

And $$a=a_{1}.$$

We create an invertible matrix denoted as

$$\begin{aligned} {\mathcal {M}}=\left( \begin{array}{cc} {\mathcal {E}}_{12} &{} {\mathcal {E}}_{12} \\ -1-{\mathcal {E}}_{11} &{} {\mathcal{ R}}_{2}-{\mathcal {E}}_{11} \end{array} \right) . \end{aligned}$$

Let

44 $$\begin{aligned} \left( \begin{array}{c} x \\ \\ y \end{array} \right) = {\mathcal {M}} \left( \begin{array}{c} {\check{x}} \\ \\ {\check{y}} \end{array} \right) , \end{aligned}$$

for (42), then the map (42) becomes

45 $$\begin{aligned} \left( \begin{array}{c} x \\ \\ y \end{array} \right) \rightarrow \left( \begin{array}{cc} -1 &{} 0 \\ &{} \\ 0 &{} {\mathcal{ R}}_{2} \end{array} \right) \left( \begin{array}{c} {\check{x}} \\ \\ {\check{y}} \end{array} \right) +\left( \begin{array}{c} f(u,v,a_{*}) \\ \\ g(u,v,a_{*}) \end{array} \right) , \end{aligned}$$

where

46 $$\begin{aligned} \begin{array}{l} f(u,v,h_{1})=\dfrac{ ( {\mathcal{ R}}_{{2}}-{\mathcal {E}}_{{11}}) {\hat{P}}_{{2}}}{{\mathcal {E}}_{{12}} ( {\mathcal{ R}}_{{2}}+1 ) }a{u}^{2}+\dfrac{ ({\mathcal{ R}}_{{2}}-{\mathcal {E}}_{{11}}) {\mathcal {E}}_{{14}}}{{\mathcal {E}}_{{12}} ({\mathcal{ R}}_{{2}}+1)}{u}^{2} - {\dfrac{{\mathcal {E}}_{{24}}}{{\mathcal{ R}}_{{2}}+1}}{v}^{2}+{\dfrac{ \left( {\mathcal {E}}_{{13}} \left( {\mathcal{ R}}_{{2}}-{\mathcal {E}}_{{11}} \right) -{\mathcal {E}}_{{23}}{\mathcal {E}}_{{12}} \right) }{{\mathcal {E}}_{{12}} \left( {\mathcal{ R}}_{{2}}+1 \right) }}vu \\ \quad \qquad \qquad + \dfrac{ \left( {\mathcal{ R}}_{{2}}-{\mathcal {E}}_{{11}} \right) {\hat{P}}_{{1}}}{{\mathcal {E}}_{{12}} \left( {\mathcal{ R}}_{{2}}+1 \right) }au +o((\left| u\right| +\left| v\right| +\left| a_{1}\right| )^{4}). \end{array}\nonumber \\ \begin{array}{l} g(u,v,h_{1})={\dfrac{ \left( 1+{\mathcal {E}}_{{11}} \right) {\hat{P}}_{{2}}}{{\mathcal {E}}_{{12} } \left( {\mathcal{ R}}_{{2}}+1 \right) }}a{u}^{2}+{\dfrac{ \left( 1+{\mathcal {E}}_{{11}} \right) {\mathcal {E}}_{{14}}}{{\mathcal {E}}_{{12}} \left( {\mathcal{ R}}_{{2}}+1 \right) }}{u}^{2}+{\dfrac{{\mathcal {E}}_{{24}}}{{\mathcal{ R}}_{{2}}+1}}{v}^{2}+{\dfrac{\left( ({\mathcal {E}}_{{11}}+1){\mathcal {E}}_{{13}}+{\mathcal {E}}_{{23}}{\mathcal {E}}_{{12}} \right) }{{\mathcal {E}}_{{12}} \left( {\mathcal{ R}}_{{2}}+1 \right) }}uv \\ \quad \qquad \qquad +{\dfrac{ \left( 1+{\mathcal {E}}_{{11}} \right) {\hat{P}}_{{1}}}{{\mathcal {E}}_{{12}} \left( {\mathcal{ R}}_{{2}}+1 \right) }}au +o((\left| u\right| +\left| v\right| +\left| a_{1}\right| )^{4}). \end{array} \end{aligned}$$

By introducing the variables $u = {\mathcal {E}}_{12}{\check{x}}+{\mathcal {E}}_{12}{\check{y}}$ and $v=-(1+{\mathcal {E}}_{11}){\check{x}}+({\mathcal {R}}_{2}-{\mathcal {E}}_{11}){\check{y}}$. Applying the center manifold theorem^27^, we obtain:

$$\begin{aligned} c_{{1}}={\dfrac{ \left( \left( {\mathcal {E}}_{{13}}-{\mathcal {E}}_{{24}} \right) {\mathcal {E}}_{{11}}+ \left( {\mathcal {E}}_{{23}}-{\mathcal {E}}_{{14}} \right) {\mathcal {E}}_{{12 }}+{\mathcal {E}}_{{13}}-{\mathcal {E}}_{{24}} \right) \left( 1+{\mathcal {E}}_{{11}} \right) }{{{\mathcal{ R}}_{{2}}}^{2}-1}},c_{{2}}=-{\dfrac{ \left( 1+{\mathcal {E}}_{{11}} \right) {\hat{P}}_{{1}}}{ \left( {\mathcal{ R}}_{{2}}+1 \right) ^{2}}}, c_{3}=0. \end{aligned}$$

Using the same technique used with the other fixed points, the center manifold map is generated as follows:

$$\begin{aligned} F:{\bar{\varkappa }}\rightarrow -{\bar{\varkappa }}+s_{1}{\bar{\varkappa }}^{2}+s_{2}{\bar{\varkappa }}a_{*}+s_{3} {\bar{\varkappa }}^{2}a_{*}+s_{4}{\bar{\varkappa }}a_{*}^{2}+s_{5}{\bar{\varkappa }}^{3}+O((|{\bar{\varkappa }} |+|a_{*}|^{4}), \end{aligned}$$

where

$$\begin{aligned} \begin{array}{l} s_{1}=\frac{1}{{\mathcal{ R}}_{2}+1}(\left( {\mathcal {E}}_{{13}}-{\mathcal {E}}_{{24}} \right) {{\mathcal {E}}_{{11}}}^{2}+ \left( \left( {\mathcal {E}}_{{23}}-{\mathcal {E}}_{{14}} \right) {\mathcal {E}}_{{12}}+ \left( 1-{\mathcal{ R}}_{{2}} \right) {\mathcal {E}}_{{13}}-2\,{\mathcal {E}}_{{24}} \right) {\mathcal {E}}_{{11}} \\ \qquad + \left( {\mathcal {E}}_{{14}}{\mathcal{ R}}_{{2}}+{\mathcal {E}}_{{23}} \right) {\mathcal {E}}_{{12}}-{\mathcal{ R}}_{{2}}{\mathcal {E}}_{{13}}-{\mathcal {E}}_{{24}} ), \\ \\ s_{2}=\frac{1}{{\mathcal{ R}}_{{2}}+1}(\left( {\mathcal{ R}}_{{2}}-{\mathcal {E}}_{{11}} \right) {\hat{P}}_{{1}}), \\ s_{3}= \frac{1}{{\mathcal{ R}}_{{2}}+1} \left( 6\,a{\hat{P}}_{{2}}+4\,{\mathcal {E}}_{{14}}-4\,{\mathcal {E}}_{{23}} \right) {\mathcal {E}}_{{12}}+4\, \left( -{\mathcal {E}}_{{24}}+{\mathcal {E}}_{{13}} \right) \left( k_{{2}}-{\mathcal {E}}_{{11}} \right) ^{2}a{c_{{2}}}^{2} \\ \qquad + ( \left( 4\,{\mathcal {E}}_{{13}} -4\,{\mathcal {E}}_{{24}} \right) {{\mathcal {E}}_{{ 11}}}^{2}+ \left( \left( 4\,{\mathcal {E}}_{{24}}-6\,{\mathcal {E}}_{{13}} \right) {\mathcal{ R}}_{{2}}+ \left( 4\,{\mathcal {E}}_{{23}}-8\,a{\hat{P}}_{{2}}-4\,{\mathcal {E}}_{{14}} \right) {\mathcal {E}}_{{12}}-4\,{\mathcal {E}}_{{24}}+2\,{\mathcal {E}}_{{13}} \right) {\mathcal {E}}_{{11}} \\ \qquad + 2\, {{\mathcal{ R}}_{{2}}}^{2}{\mathcal {E}}_{{13}}+ \left( \left( 8\,a{\hat{P}}_{{2}}+4\,{\mathcal {E}}_{ {14}}-2\,{\mathcal {E}}_{{23}} \right) {\mathcal {E}}_{{12}}+4\,{\mathcal {E}}_{{24}}-2\, {\mathcal {E}}_{{13}} \right) {\mathcal{ R}}_{{2}}+2\,{\mathcal {E}}_{{12}}{\mathcal {E}}_{{23}}) c_ {{2}} \\ \qquad + 2\, \left( {\mathcal{ R}}_{{2}}-{\mathcal {E}}_{{11}} \right) \left( {\mathcal {E}}_{{12}}{\hat{P}}_{{2}}+c_{{1}}{\hat{P}}_{{1}} \right) , \end{array} \end{aligned}$$

$$\begin{aligned} \begin{array}{l} s_{4}=\frac{1}{{\mathcal{ R}}_{{2}}+1} (2\,\left( {\mathcal{ R}}_{{2}}-{\mathcal {E}}_{{11}} \right) c_{{2}}{\hat{P}} _{{1}}),\\ s_{5}=\frac{1}{{\mathcal{ R}}_{{2}}+1}(( 2\,{\mathcal {E}}_{{13}}-2\,{\mathcal {E}}_{ {24}}) {{\mathcal {E}}_{{11}}}^{2}+ (( 2 \,{\mathcal {E}}_{{24}}-3\,{\mathcal {E}}_{{13}}) {\mathcal{ R}}_{{2}}+( 2\,{\mathcal {E}}_{{ 23}}-2\,{\mathcal {E}}_{{14}}) {\mathcal {E}}_{{12}}+{\mathcal {E}}_{{13}}-2\,{\mathcal {E}}_{{24}}) {\mathcal {E}}_{{11}} \\ \qquad +{{\mathcal{ R}}_{{2}}}^{2}{\mathcal {E}}_{{13}}+ \left( \left( 2\,{\mathcal {E}}_{{ 14}}-{\mathcal {E}}_{{23}} \right) {\mathcal {E}}_{{12}}-{\mathcal {E}}_{{13}}+2\,{\mathcal {E}}_{{24} } \right) {\mathcal{ R}}_{{2}}+{\mathcal {E}}_{{12}}{\mathcal {E}}_{{23}}) c_{{1}} . \end{array} \end{aligned}$$

Let

$$\begin{aligned} \Psi _{1}=\left( \frac{\partial ^{2}F}{\partial {\bar{\varkappa }}\partial a_{*}}+ \frac{1}{2}\frac{\partial F}{\partial a{*}}\frac{\partial ^{2}F}{ \partial {\bar{\varkappa }}^{2}}\right) |_{\left( 0,0\right) }=s_{2,} \end{aligned}$$

and

$$\begin{aligned} \Psi _{2}=\left( \frac{1}{6}\frac{\partial ^{3}F}{\partial {\bar{\varkappa }}^{3}}+\left( \frac{1}{2}\frac{\partial ^{2}F}{\partial {\bar{\varkappa }}^{2}}\right) ^{2}\right) |_{\left( 0,0\right) }=s_{1}^{2}+s_{5}. \end{aligned}$$

Based on the preceding discussion, we can state the following theorem:

#### Theorem 11

*If* $\Psi _{1}\ne 0$ and $\Psi _{2}\ne 0$, then model (1) *undergoes a period-doubling bifurcation at the unique* *positive fixed point* $P_{3}({\check{x}}, {\check{y}})$ *when the parameter* *a* *varies within a small neighborhood of* $F_{P_{3}}$. *Additionally, when* $\Psi _{2}>0$ (*respectively*, $\Psi _{2}<0$), *the period-2 orbits that arise from* $P_{3}({\check{x}}, {\check{y}})$ *exhibit stability* (*respectively, instability*).

Now, let’s examine the Neimark-Sacker bifurcation of $P_{3}({\check{x}}, {\check{y}})$ when the parameters (*a*, *b*, *c*, *d*) vary within a small neighborhood of $N_{P_{3}}$. By selecting parameters $(a, b, c, d) \in N_{P_{3}}$, we can represent the model (1) as follows:

47 $$\begin{aligned} \left\{ \begin{array}{l} x_{n+1}=a_{2}x_{n}(1-x_{n})-cx_{n}y_{n}, \\ y_{n+1}=by_{n}(1-y_{n})+dx_{n}y_{n}. \\ \end{array} \right. \end{aligned}$$

We have parameter $(a, b, c, d) \in N_{P_{3}}$, then $a_{2}={\dfrac{K \pm \sqrt{{K}^{2}-4\,L}}{2\,b \left( b+d-2 \right) }}$. Choosing $a_{2}$ as a bifurcation parameter and little disturb the model (47) by ${\bar{a}}_{*}\ll 1$ to get

48 $$\begin{aligned} \left\{ \begin{array}{l} x_{n+1}=(a_{2}+{{\bar{a}}}_{*})x_{n}(1-x_{n})-cx_{n}y_{n}, \\ y_{n+1}=by_{n}(1-y_{n})+dx_{n}y_{n}. \\ \end{array} \right. \end{aligned}$$

Let $u=x-{\check{x}}$, $u=y-{\check{y}}$. Then model (47) converted to

49 $$\begin{aligned} \left( \begin{array}{c} {u} \\ \\ {v} \end{array} \right) {\tiny \rightarrow }\left( \begin{array}{c} {\hat{E}}_{11}u+{\hat{E}}_{12}v+{\hat{E}}_{13}uv+{\hat{E}}_{14}u^{2} \\ \\ {\hat{E}}_{21}u+{\hat{E}}_{22}v+{\hat{E}}_{23}uv+{\hat{E}}_{24}v^{2} \\ \end{array} \right) , \end{aligned}$$

the values of ${\hat{E}}_{11}, {\hat{E}}_{12}, {\hat{E}}_{13}, {\hat{E}}_{14}, {\hat{E}}_{21}, {\hat{E}}_{22}, {\hat{E}}_{23}, {\hat{E}}_{14}$ are obtained from Eq. (43) by substituting *a* with $a_{2}+{\bar{a}}_{*}$. The characteristic equation of the Jacobian matrix evaluated at $(u,v) = (0, 0)$ can be written as:

$$\begin{aligned} {\mathcal{ R}}^{2}+p({\bar{a}}_{*}){\mathcal{ R}}+q({\bar{a}}_{*})=0, \end{aligned}$$

where

$$\begin{aligned} p({\bar{a}}_{*})= \left( 1-2\,x \right) (a_{2}+{{\bar{a}}}_{*})-cy+ \left( 1-2\,y \right) b+dx, \end{aligned}$$

and

$$\begin{aligned} q({\bar{a}}_{*})= \left( \left( 2\,y-1 \right) b-dx \right) \left( 2\,x-1 \right) (a_{2}+{{\bar{a}}}_{*})+ \left( 2\,y-1 \right) cby. \end{aligned}$$

As the parameters (*a*, *b*, *c*, *d*) belong to the neighborhood $N_{P_{3}}$, the eigenvalues of $p_{3}({\check{x}}, {\check{y}})$ can be expressed as a pair of complex conjugate numbers ${\mathcal{ R}}$ and $\overline{\mathcal{R}}$, with a modulus given by Eq. (1) according to Theorem 4. Here, we have:

$$\begin{aligned} {\mathcal{ R}},\overline{\mathcal {R}} = & {} \frac{p({{\bar{a}}}_{*})}{2}\pm i\frac{\sqrt{4q({{\bar{a}}}_{*})-p^{2}({{\bar{a}}}_{*})}}{2}. \end{aligned}$$

Then we get

$$\begin{aligned} \left| {\mathcal{ R}}\right| =\sqrt{q({{\bar{a}}}_{*})},\ell =\frac{d\left| {\mathcal{ R}}\right| }{d{{\bar{a}}}_{*}}|_{\bar{a}_{*}=0}={\dfrac{\beta \, \left( \alpha \,b+d\delta \right) }{2}}>0. \end{aligned}$$

In addition, when ${\bar{a}}_{*}=0$, , ${\mathcal{ R}}^{\theta }$, $\overline{\mathcal{R}} ^{\theta }\ne 1$, $\theta =1,$ 2,  3,  4, which is equivalent to $p(0)\ne -2,$ 0,  1,  2. This leads to

50 $$\begin{aligned} bcy \left( 1-2\,y \right) =1 \ne 1, 2. \end{aligned}$$

Next, we analyze the normal form of Eq. (49) when ${\bar{a}}_{*}=0$. Let’s define:

$$\begin{aligned} m=\frac{a\left( \left( c-d+4 \right) b-c-{b}^{2} \right) +d \left( b+2\,c \right) -{a}^{2}b}{2(ab + cd)}, \end{aligned}$$

and

$$\begin{aligned} \omega =\frac{\sqrt{\phi +\psi }}{2(ab+cd)}, \end{aligned}$$

where $$\phi = 2\,b ( {b}^{2}+ ( c+d) b-c ) {a}^{3}+ ( -{b}^{4}+ ( -2\,c-2\,d ) {b}^{3}- ( c-d ) (c-d-2) {b}^{2} +2\,c \left( 2\,{d}^{2}+c-d \right) b-{c}^{2}) {a}^{2}-{a}^{4}{b}^{2}$$,

$$\psi =2\,d \left( {b}^{3}+ \left( -2\,{c}^{2}-c+d \right) {b}^{2}- \left( 2 \,c \left( d-2 \right) +4\,d-1 \right) cb+2\,{c}^{2} \left( d-1 \right) \right) a-{d}^{2} \left( {b}^{2}+ \left( -4\,{c}^{2}-4\,c \right) b+4\,{c}^{2} \right)$$.

And use an appropriate change in coordinates to transform the model (49) into the following form:

51 $$\begin{aligned} \left( \begin{array}{c} {\check{u}} \\ \\ {\check{v}} \end{array} \right) \rightarrow \left( \begin{array}{cc} m &{} -\omega \\ &{} \\ \omega &{} m \end{array} \right) \left( \begin{array}{c} {\check{u}} \\ \\ {\check{v}} \end{array} \right) +\left( \begin{array}{c} f({\check{u}},{\check{v}},h_{*}) \\ \\ g({\check{u}},{\check{v}},h_{*}) \end{array} \right) , \end{aligned}$$

where

$$\begin{aligned} \begin{array}{l} {\check{f}}({\check{u}},{\check{v}},h_{*})=\dfrac{1}{{\hat{E}}_{{12}}} ({\hat{E}}_{{14}}{u}^{2}+{\hat{E}}_{{13}}uv), \end{array} \end{aligned}$$

and

$$\begin{aligned} \begin{array}{l} {\check{g}}({\check{u}},{\check{v}},h_{*})={\dfrac{{\hat{E}}_{{14}} \left( m-{\hat{E}}_{{11}} \right) {u}^{2}}{{\hat{E}}_{{12}}\omega }}+{\dfrac{ \left( \left( m-{\hat{E}}_{{11}} \right) {\hat{E}}_{{13}}-{\hat{E}}_{{23}}{\hat{E}}_{{12}} \right) uv}{{\hat{E}}_{{12}}\omega }}-{\dfrac{{\hat{E}}_{{12}}{\hat{E}}_{{24}}{v}^{2}}{{\hat{E}}_{{12}}\omega }}. \end{array} \end{aligned}$$

In addition,

$$\begin{aligned}{} & {} \begin{array}{ccc} {\check{f}}_{{\check{u}}{\check{u}}}=2((m-{\hat{E}} _{11}){\hat{E}} _{13}+{\hat{E}} _{12}{\hat{E}} _{14}),&\quad{\check{f}}_{{\check{u}}{\check{v}}}=-\omega {\hat{E}} _{13},&\quad{\check{f}}_{{\check{v}}{\check{v}}}=0, \end{array} \qquad \qquad \quad \quad \\{} & {} \\{} & {} \begin{array}{cccc} {\check{f}}_{{\check{u}}{\check{u}}{\check{u}}}=0,&\quad{\check{f}}_{{\check{u}}{\check{u}}{\check{v}}}=0,&\quad{\check{f}}_{{\check{u}}{\check{v}}{\check{v}}}=0,&\quad{\check{f}} _{{\check{v}}{\check{v}}{\check{v}}}=0, \end{array} \end{aligned}$$

and

$$\begin{aligned} \begin{array}{l} {\check{g}}_{{\check{u}}{\check{u}}}={\dfrac{2}{\omega } \left( m-{\hat{E}}_{{11}} \right) \left( \left( {\hat{E}}_{{24}}-{\hat{E}}_{{13}} \right) {\hat{E}}_{{11}}+ \left( {\hat{E}}_ {13}- {\hat{E}}_{24} \right) m+{\hat{E}}_{{12}} \left( {\hat{E}}_{{14}}-{\hat{E}}_{{23}} \right) \right) }, \\ \\ {\check{g}}_{{\check{u}}{\check{v}}}= \left( {\hat{E}}_{{13}}-2\,{\hat{E}}_{{24}} \right) {\hat{E}}_{{11}}-m{\hat{E}}_{{13}}+2\,m{\hat{E}}_{{24}}+{\hat{E}}_{{23}}{\hat{E}}_{{12}}, \\ \\ {\check{g}}_{{\check{v}}{\check{v}}}=-2\,\omega \,{\hat{E}}_{{24}}, \begin{array}{cccc} {\check{g}}_{{\check{u}}{\check{u}}{\check{u}}}=0, &{} {\check{g}}_{{\check{u}}{\check{u}}{\check{v}}}=0, &{} {\check{g}}_{{\check{u}}{\check{v}}{\check{v}}}=0, &{} {\check{g}} _{{\check{v}}{\check{v}}{\check{v}}}=0. \end{array} \end{array} \end{aligned}$$

When the discriminant quantity below is non-zero, the map (51) can experience the Neimark–Sacker bifurcation:

$$\mathfrak{f} =Re\bigg[\frac{(1-2{\mathcal{ R}}) \bar{{\mathcal{ R}}}^{2}}{1-{\mathcal{ R}}}\Phi_{11} \Phi_{20} \bigg]+ \frac{1}{2}\left|\Phi_{11} \right|^{2}+\left|\Phi_{02} \right|^{2}-Re(\bar{{\mathcal{ R}}} \Phi_{21}),$$

where

$$\begin{aligned} \Phi _{20}= & {} \frac{1}{8}[{\check{f}}_{{\check{u}}{\check{u}}}-{\check{f}}_{{\check{v}}{\check{v}}}+2{\check{g}}_{{\check{u}}{\check{v}}}+i( {\check{g}}_{{\check{u}}{\check{u}}}-{\check{g}}_{{\check{v}}{\check{v}}}-2{\check{f}}_{{\check{u}}{\check{v}}})], \\ \Phi _{11}= & {} \frac{1}{4}[{\check{f}}_{{\check{u}}{\check{u}}}+{\check{f}}_{{\check{v}}{\check{v}}}+i({\check{g}}_{{\check{u}}{\check{u}}}+ {\check{g}}_{{\check{v}}{\check{v}}})], \\ \Phi _{02}= & {} \frac{1}{8}[{\check{f}}_{{\check{u}}{\check{u}}}-{\check{f}}_{{\check{v}}{\check{v}}}-2{\check{g}}_{{\check{u}}{\check{v}}}+i( {\check{g}}_{{\check{u}}{\check{u}}}-{\check{g}}_{{\check{v}}{\check{v}}}+2{\check{f}}_{{\check{u}}{\check{v}}})], \\ \Phi _{21}= & {} \frac{1}{16}[{\check{f}}_{{\check{u}}{\check{u}}{\check{u}}}+{\check{f}}_{{\check{u}}{\check{v}}{\check{v}}}+{\check{g}} _{{\check{u}}{\check{u}}{\check{v}}}+{\check{g}}_{{\check{v}}{\check{v}}{\check{v}}}+i({\check{g}}_{{\check{u}}{\check{u}}{\check{u}}}+ {\check{g}}_{{\check{u}}{\check{v}}{\check{v}}}-{\check{f}}_{{\check{u}}{\check{u}}{\check{v}}}- {\check{f}}_{{\check{v}}{\check{v}}{\check{v}}})]. \end{aligned}$$

#### Theorem 12

*If condition* (50)* is satisfied and* ${\mathfrak{f}}$≠ 0,* model* (1)* undergoes a Neimark–Sacker bifurcation at the fixed point* $p_{3}({\check{x}}, {\check{y}})$ *when the parameter* *a varies within a small neighborhood of* $N_{p_{3}}$.* Additionally, if* ${\mathfrak{f}}$ < 0 (*respectively*, ${\mathfrak{f}}$> 0),* an attracting (respectively, repelling) invariant closed curve bifurcates from the fixed point for* $a>a_{2}$ (*respectively*, $a<a_{2}$).

## Existence of Marotto’s chaos

In this section, we establish that the model (1) displays chaotic behavior, as defined by Marotto^31,32^.

### Definition 2

(42) Let’s consider the function ${\mathcal {F}}:{\mathbb {R}}^{\mu } \rightarrow {\mathbb {R}}^{\mu }$, which is differentiable in the neighborhood $B_{r}(Z)$. We define $Z \in {\mathbb {R}}$ as an expanding fixed point of ${\mathcal {F}}$ in $B_{r}(Z)$ if ${\mathcal {F}}(Z) = Z$ and all eigenvalues of the Jacobian matrix $D{\mathcal {F}}({\mathcal {X}})$ have a magnitude greater than 1 for all ${\mathcal {X}} \in B_{r}(Z)$.

### Definition 3

(42) Suppose Z is an expanding fixed point of ${\mathcal {F}}$ in $B_{r}(Z)$ for a positive value r. In such a case, Z is considered a snapback repeller of ${\mathcal {F}}$ if there exists a point ${\mathcal {X}}_{0}\in B_{r}(Z)$ with ${\mathcal {X}}_{0} = Z$, ${\mathcal {F}}^{\eta }({\mathcal {X}}_{0}) \ne Z$, and $D{\mathcal {F}}^{\eta }({\mathcal {X}}_{0}) \ne 0$ for a positive integer $\eta$.

Firstly, we establish the condition under which $P_{3}$ is identified as an expanding fixed point of the model (1), as follows:

52 $$\begin{aligned} \begin{array}{c} {\mathcal {F}}({\mathcal {X}}_{n}) \end{array} {=}\left( \begin{array}{c} ax_{n}(1-x_{n})-cx_{n}y_{n} \\ by_{n}(1-y_{n})+dx_{n}y_{n} \end{array} \right) , \qquad {\mathcal {X}}_{n}=(x_{n}\qquad y_{n})^T. \end{aligned}$$

The eigenvalues associated with the fixed point $p_{3}({\check{x}}, {\check{y}})$ can be determined as follows:

$$\begin{aligned} {\mathcal{ R}},\overline{\mathcal{R}} = & {} \frac{p({\check{x}}, {\check{y}})}{2}\pm \frac{\sqrt{p^{2}({\check{x}}, {\check{y}})-4q({\check{x}}, {\check{y}})}}{2}, \end{aligned}$$

where

$$p({\check{x}}, {\check{y}}) = \left( d -2\,a \right) x-\left( 2\,b+c \right) y +a+b$$,

and $$q({\check{x}}, {\check{y}}) = \left( 2\,ax-a \right) \left( \left( 2\,y-1 \right) b-dx \right) +b cy \left( 2\,y-1 \right)$$.

Let’s assume that the eigenvalues associated with the fixed point $p_{3}({\check{x}}, {\check{y}})$ are a pair of complex eigenvalues ${\mathcal{ R}}$ and $\overline{\mathcal{R}}$, and their norms exceed unity. These conditions are equivalent to

$$\begin{aligned} \left\{ \begin{array}{l} p^{2}({\check{x}}, {\check{y}})<4q({\check{x}}, {\check{y}}), \\ q({\check{x}}, {\check{y}})>1.\\ \end{array} \right. \end{aligned}$$

Let

$$\mathcal S_{1}(x, y) = \left( \left( 2\,b+c \right) y+ \left( 2\,a-d \right) x-a-b \right) ^{2}-4\, \left( 2\,ax-a \right) \left( \left( 2\,y-1 \right) b-dx \right) -4\,bcy \left( 2\,y-1 \right)$$,

if $\hbox {y} > 0$, then for

$$\left( 2\,x-1 \right) ^{2}{a}^{2}- \left( 4\,x-2 \right) \left( \left( 2\,y-1 \right) b-cy-dx \right) a+ \left( 2\,y-1 \right) ^{2}{b }^{2}- \left( 4\,y-2 \right) \left( cy+dx \right) b+ \left( cy-dx \right) ^{2}<0$$,

we have

$$x_{1}=\frac{A-\sqrt{B}}{ \left( d+2\,a \right) ^{2}}<x<\frac{A+\sqrt{B}}{ \left( d+2\,a \right) ^{2}}=x_{2}$$,

where

$$A=2\,{a}^{2}+ a \left( d+ \left( 4\,b-2\,c \right) y-2\,b \right) + d \left( \left( 2\,b+c \right) y-b \right)$$,

and

$$B=4ycd\, \left( 2\,{a}^{2}+ \left( \left( 4\,y-2 \right) b-2\,cy+d \right) a+db \left( 2\,y-1 \right) \right)$$.

Thus, $$\mathcal S_{1}(x, y)<0 \; if \; x \in E_{1}=(x_{1}\bigcup x_{2}) = \{x|\frac{A-\sqrt{B}}{ \left( d+2\,a \right) ^{2}}<x<\frac{A+\sqrt{B}}{ \left( d+2\,a \right) ^{2}}\}\; and \; y > 0$$.

Let

$$\mathcal S_{2}(x, y)=\left( 2\,x-1 \right) \left( \left( 2\,y-1 \right) b-dx \right) a-1 +bcy \left( 2\,y-1 \right)$$,

if $\hbox {y} > 0$, then for

$$\left( 2\,ax-a \right) \left( \left( 2\,y-1 \right) b-dx \right) +b cy \left( 2\,y-1 \right) -1>0$$,

we have

$$x_{3}=\frac{C-\sqrt{D}}{ 4ad}<x<\frac{C+\sqrt{D}}{4ad}=x_{4}$$,

where

$$C= \left( \left( 4\,y-2 \right) b+d \right) a$$,

and

$$D={a}^{2} \left( \left( 4\,y-2 \right) b-d \right) ^{2}+8\,a \left( 2\,bc{y}^{2}-bcy-1 \right) d$$.

Thus, $$\mathcal S_{1}(x, y)<0 \; if \; x \in E_{2}=(-\infty ,x_{3})\bigcup (x_{4},\infty ) = \{x|\frac{C-\sqrt{D}}{ 4ad}<x<\frac{C+\sqrt{D}}{4ad}\}\; and \; y > 0$$.

Drawing upon the analysis mentioned above, we arrive at the following lemma:

### Lemma 2

*Let* $b \left( c+1 \right) <ab+c$ *and* $d+a<a \left( b+d \right)$, *if* $x \in E_{1} \bigcap E_{2}$ *and* $\hbox {y}>0$, *then* $p^{2}({\check{x}}, {\check{y}})-4q({\check{x}}, {\check{y}})<0$ *and* $q({\check{x}}, {\check{y}})-1>0$. Furthermore, it the fixed point $\hat{\mathsf z}({\check{x}}, {\check{y}})$ *of system* (1) *meets the following condition*:

$\hat{\mathsf z}({\check{x}}, {\check{y}}) \in U_{\hat{\mathsf z}}=\{ (x, y)\mid x \in E_{1} \bigcap E_{2},y>0 \}$, then $\hat{\mathsf z}({\check{x}}, {\check{y}})$ is an expanding fixed point in $U_{\hat{\mathsf z}}$.

According to the definition of a snapback repeller point, we should identify point $z_{1}(x_{1}, y_{1}) \in U_{\hat{\mathsf z}}$ such that $z_{1}\ne \hat{\mathsf z}$, ${\mathcal {F}}^{\eta }(z_{1}) = \hat{\mathsf z}$.

$\left| { D {\mathcal {F}}} ^{\eta } \left( z_{{1}} \right) \right| \ne 0$, for a positive integer $\eta$, where Map ${\mathcal {F}}$ is defined by (1).

To continue, observe that

53 $$\begin{aligned} \left\{ \begin{array}{l} ax_{1}(1-x_{1})-cx_{1}y_{1}=x_{2}, \\ by_{1}(1-y_{1})+dx_{1}y_{1}=y_{2}, \\ \end{array} \right. \end{aligned}$$

and

54 $$\begin{aligned} \left\{ \begin{array}{l} ax_{2}(1-x_{2})-cx_{2}y_{2}={\check{x}}, \\ by_{2}(1-y_{2})+dx_{2}y_{2}={\check{y}}. \\ \end{array} \right. \end{aligned}$$

We have defined a second iteration map, denoted as ${\mathcal {F}}^{2}$, which transforms the point $z_{1}(x_{1}, y_{1})$ into the fixed point $\hat{\mathsf z}({\check{x}},{\check{y}})$, assuming that there are solutions other than $\hat{\mathsf z}$ for Eqs. (53) and (54). These additional solutions for Eq. (54) satisfy the following equation:

55 $$\begin{aligned} \left\{ \begin{array}{l} x_{{2}}={\frac{1}{dy_{{2}}}(b{y_{{2}}}^{2}-by_{{2}}+{\check{y}})} , \\ y_{{2}}=y_{{2}}+{\check{x}}+ \left( {\frac{1}{{d}^{2}{y_{{2}}}^{2}}} \right) \left( b{y_{{2}}}^{2}-by_{{2}}+{\check{y}} \right) \left( \left( ab+cd \right) {y_{{2}}}^{2}-a \left( b+d \right) y_{{2}}+a{\check{y}} \right) . \\ \end{array} \right. \end{aligned}$$

By substituting $x_{{2}}$ and $y_{{2}}$ into Eq. (53) and solving for $x_{{1}}$ and $y_{{1}}$, we obtain

56 $$\begin{aligned} \left\{ \begin{array}{l} x_{{1}}={\frac{1}{dy_{{1}}}(b{y_{{1}}}^{2}-by_{{1}}+y_{{2}})} , \\ y_{{1}}=y_{{1}}+x_{{2}}+ \left( {\frac{1}{{d}^{2}{y_{{1}}}^{2}}} \right) \left( b{y_{{1}}}^{2}-by_{{1}}+y_{{2}} \right) \left( \left( ab+cd \right) {y_{{1}}}^{2}-a \left( b+d \right) y_{{1}}+ay_{{2}} \right) . \\ \end{array} \right. \end{aligned}$$

Through straightforward calculations, we obtain

$$\left| { D {\mathcal {F}}} ^{2} \left( z_{{1}} \right) \right| =\left( a \left( 2\,B-1 \right) \left( \left( 2\,C-1 \right) b-Bd \right) +Cbc \left( 2\,C-1 \right) \right) \left( cdxy-a\delta \,x+A \delta \right) ,$$

where

$$A:=a \left( 1-x \right) -cy, B:=ax \left( 1-x \right) -cxy, C:=dxy+b \left( 1-y \right) y\;and\;\delta :=dx+b \left( 1-y \right) -by.$$

Clearly, when the condition in Lemma 2 holds, $z_{1}(x_{1}, y_{1})$, $z_{2}(x_{2}, y_{2})\ne$ $\hat{\mathsf z}({\check{x}},{\check{y}})$, $z_{1}(x_{1}, y_{1})\in U_{\hat{\mathsf z}}$ and $\left| { D {\mathcal {F}}} ^{2} \left( z_{{1}} \right) \right| \ne 0$, In that case, $\hat{\mathsf z}$ qualifies as a snap-back repeller within $U_{\hat{\mathsf z}}$. Consequently, the following theorem is confirmed:

### Theorem 13

*Suppose the conditions outlined in Lemma* 2*are met. If*

(1) $$\left( 2\,x-1 \right) ^{2}{a}^{2}- \left( 4\,x-2 \right) \left( \left( 2\,y-1 \right) b-cy-dx \right) a+ \left( 2\,y-1 \right) ^{2}{b }^{2}- \left( 4\,y-2 \right) \left( cy+dx \right) b+ \left( cy-dx \right) ^{2}<0$$ and $$\left( 2\,ax-a \right) \left( \left( 2\,y-1 \right) b-dx \right) +b cy \left( 2\,y-1 \right) -1>0$$.

(2) If the solutions $(x_{2}, y_{2})$ and $(x_{1}, y_{1})$ of Eqs. (55) and (56) satisfy the additional conditions that $(x_{1}, y_{1})$, $(x_{2}, y_{2})$ $\ne ({\check{x}}, {\check{y}})$, $(x_{1}, y_{1}) \in U_{\hat{\mathsf z}}$, $(x_{1}, y_{1})\ne (0, 0)$, and $\left| { D {\mathcal {F}}} ^{2} \left( z_{{1}} \right) \right| \ne 0$, then $\hat{\mathsf z}({\check{x}}, {\check{y}})$ qualifies as a snap-back repeller for model (1). Consequently, model (1) exhibits chaos in accordance with Marotto’s definition.

Next, an example is provided to demonstrate how to fulfil the conditions outlined in Theorem 13.

### Example 1

Consider the values of the parameters $a = 3.5, b = 3, c = 1.25$ and $d = 1.5$ in the collection $(\delta , b) \in \{ (\delta , b), b>{\frac{\delta + \sqrt{{\delta }^{2}-4\,\theta }}{2\,a ( a -c-2 ) }} )\}$, where $\delta := \left( -d+2 \right) {a}^{2}+ \left( \left( c+3 \right) d-3\,c-3 \right) a+ \left( -c-2 \right) d$, $\theta :=a \left( a-c-2 \right) \left( \left( d-2 \right) a-d \right) c$, we have $({\check{x}}, {\check{y}})=(0.404040404, 0.8686868687)$, and the eigenvalues linked to this point are denoted as $\lambda =- 1.010101010 \pm 0.5503892037 \,i$. We are able to identify a point $(x_{1}, y_{1})=(0.08203563254, 0.8608928048)$, which satisfies ${\mathcal {F}} ^{2}(x_{1}, y_{1})=({\check{x}}, {\check{y}})$ and $\left| { D {\mathcal {F}}} ^{2} \left( z_{{1}} \right) \right| \ne 0$ therefore $({\check{x}}, {\check{y}})$ is a snap-back repeller.

## Chaos control

The utilisation of different bifurcation parameters enhances our ability to comprehensively analyse the system’s behavior. By selecting independent parameters, we can isolate their influences and gain insights into their effects on the system’s dynamics. This approach allows us to examine the system’s sensitivity to various parameters, identify those that have a significant impact on bifurcations, and develop a deeper understanding of its behavior under diverse conditions. Choosing multiple parameters makes the study practical and feasible, as some of them may be controlled or measured in experiments. Furthermore, effective ecological conservation management solutions can be devised to preserve these intricate ecosystems. Chaotic dynamics within a system can lead to instabilities and undesirable behaviors. Therefore, effective techniques for chaos control are crucial for mitigating harmful chaotic behaviors. To improve system performance, it is necessary to regulate chaotic dynamics towards a periodic orbit or a fixed point. Chaos control involves managing chaotic dynamics within complex nonlinear systems, and the literature offers numerous strategies for achieving this, such as the Ott–Grebogi–Yorke (OGY) method^10,33,34^ and feedback control methods applied to model (1). For controlling chaos arising from Neimark–Sacker and period-doubling bifurcations at the fixed point of model (57), we employ the OGY technique, resulting in the following formulation of model (57):

57 $$\begin{aligned} \begin{array}{l} x_{n+1}=ax_{n}(1-x_{n})-cx_{n}y_{n}=f(x_{n},y_{n},c), \\ y_{n+1}=by_{n}(1-y_{n})+dx_{n}y_{n}=g(x_{n},y_{n},c). \end{array} \end{aligned}$$

We introduce the parameter *c* as a chaos control parameter, with *c* belonging to the interval $(c_{0}-\delta ,c_{0}+\delta )$, where $\delta >0$ and $c_{0}$ represents the nominal value of *c*. Additionally, we consider $p_{3}({\check{x}}, {\check{y}})$ as the fixed point of model (1). In the vicinity of the fixed point $p_{3}({\check{x}}, {\check{y}})$, model (57) can be approximated as follows:

58 $$\begin{aligned} \left[ \begin{array}{c} x_{n+1}-{\check{x}} \\ y_{n+1}-{\check{y}} \end{array} \right] \approx J({\check{x}},{\check{y}},c_{0})\left[ \begin{array}{c} x_{n}-{\check{x}} \\ y_{n}-{\check{y}} \end{array} \right] + {\mathcal{ L}}[c-c_{0}] , \end{aligned}$$

where

$$\begin{aligned} J({\check{x}},{\check{y}},c_{0})=\left[ \begin{array}{cc} \frac{\partial f({\check{x}},{\check{y}},c_{0})}{\partial x} &{} \frac{\partial f({\check{x}},{\check{y}},c_{0})}{\partial y} \\ &{} \\ \frac{\partial g({\check{x}},{\check{y}},c_{0})}{\partial x} &{} \frac{\partial g({\check{x}},{\check{y}},c_{0})}{\partial y} \end{array} \right] , \end{aligned}$$

and

$$\begin{aligned} {\mathcal{ L}}=\left[ \begin{array}{c} \frac{\partial f({\check{x}},{\check{y}},c_{0})}{\partial c} \\ \\ \frac{\partial g({\check{x}},{\check{y}},c_{0})}{\partial c} \end{array} \right] =\left[ \begin{array}{c} {\frac{ \left( \left( c+1 \right) b-c-ab \right) \left( \left( b+d -1 \right) a-d \right) }{ \left( ab+cd \right) ^{2}}} \\ \\ 0 \end{array} \right] . \end{aligned}$$

Furthermore, the controlled model (57) can be represented by the following matrix if the condition holds:

$$\begin{aligned} \tau =\left[ {\mathcal{ L}}:J{\mathcal{ L}}\right] =\left[ \begin{array}{ccc} \frac{\partial f({\check{x}},{\check{y}},c_{0})}{\partial c} &{} &{} \frac{\partial f({\check{x}},{\check{y}},c_{0})}{\partial x}\cdot \frac{\partial f({\check{x}},{\check{y}},c_{0})}{\partial c} \\ &{} &{} \\ \frac{\partial g({\check{x}},{\check{y}},c_{0})}{\partial c} &{} &{} \frac{\partial g({\check{x}},{\check{y}},c_{0})}{\partial x}\cdot \frac{\partial g({\check{x}},{\check{y}},c_{0})}{\partial c} \end{array} \right] , \end{aligned}$$

has rank 2. Since ${\frac{ \left( \left( c+1 \right) b-c-ab \right) \left( \left( b+d-1 \right) a-d \right) }{ \left( ab+cd \right) ^{2}}} \ne 0$, therefore rank of $\tau$ is 2. Next, we assume that

$$[c-c_{0}]=-\mathcal \lambda \left[ \begin{array}{c} x_{n}-{\check{x}} \\ y_{n}-{\check{y}} \end{array} \right]$$, where $$\mathcal \lambda =\left[ \begin{array}{cc} \mathcal \lambda _{1}&\mathcal \lambda _{2} \end{array} \right]$$, then system (58) can be written as

$$\begin{aligned} \left[ \begin{array}{c} x_{n+1}-{\check{x}} \\ y_{n+1}-{\check{y}} \end{array} \right] \approx \left[ J-{\mathcal{ L}} \mathcal \lambda \right] \left[ \begin{array}{c} x_{n}-{\check{x}} \\ y_{n}-{\check{y}} \end{array} \right] . \end{aligned}$$

Furthermore, the fixed point $p_{3}({\check{x}}, {\check{y}})$ is considered locally stable if and only if both eigenvalues of the matrix $J-{\mathcal {L}}\lambda$ lie within an open unit disk. The matrix $J-{\mathcal {L}}\lambda$ can be written as follows:

$$\begin{aligned} J-{\mathcal{ L}} \mathcal \lambda =\left[ \begin{array}{cc} j_{11}-\Phi \lambda _{1} &{} j_{12}-\Phi \lambda _{2} \\ &{} \\ j_{21} &{} j_{22} \end{array} \right] , \end{aligned}$$

where

$$\begin{aligned} \begin{array}{ccc} j_{11}={\frac{\left( \left( c+2 \right) b-c \right) a+cd-{a}^{2}b}{ab+cd}}, &{} j_{12}={\frac{ \left( \left( c+1-a \right) b-c \right) c}{ab+cd}},\\ &{} \\ j_{21}={\frac{d \left( \left( b+d-1 \right) a-d \right) }{ab+cd}}, &{} j_{22}={\frac{ \left( \left( 2-d \right) a+d \right) b+cd-a{b}^{2}}{ab+cd}}, \end{array} \end{aligned}$$

$$\begin{aligned} \begin{array}{c} \Phi = {\frac{ \left( \left( c+1 \right) b-c-ab \right) \left( \left( b+d-1 \right) a-d \right) }{ \left( ab+cd \right) ^{2}}}. \end{array} \end{aligned}$$

The auxiliary equation of the Jacobian matrix $J-{\mathcal {L}}\lambda$ is:

59 $$\begin{aligned} \varvec{\rho }({\mathcal {R}})={\mathcal{ R}}^{2}-(j_{11}+j_{22}-\Phi \lambda _{1}){\mathcal{ R}}+j_{22}(j_{11}-\Phi \lambda _{1})-j_{21}(j_{12}-\Phi \lambda _{2})=0 . \end{aligned}$$

Let ${\mathcal{ R}}_{1}$ and ${\mathcal{ R}}_{2}$ are the eigenvalues of characteristic equation (59), then we have

60 $$\begin{aligned} {\mathcal{ R}}_{1}+{\mathcal{ R}}_{2}=j_{11}+j_{22}-\Phi \lambda _{1}, \end{aligned}$$

and

61 $$\begin{aligned} {\mathcal{ R}}_{1}{\mathcal{ R}}_{2}=j_{{22}} \left( j_{{11}}-\Phi \,\lambda _{{1}} \right) +j_{{21}} \left( \Phi \,\lambda _{{2}}-j_{{12}} \right) . \end{aligned}$$

Furthermore, we assume ${\mathcal{ R}}_{1}=\pm 1$ and ${\mathcal{ R}}_{1}{\mathcal{ R}}_{2}=1$. Consequently, the lines of marginal stability for (60) and (61) can be calculated as follows:

62 $$\begin{aligned} {\mathcal{ L}}_{1}:j_{{22}} \left( j_{{11}}-\Phi \,\lambda _{{1}} \right) +j_{{21}} \left( \Phi \,\lambda _{{2}}-j_{{12}} \right) -1=0. \end{aligned}$$

Next, we suppose that ${\mathcal{ R}}_{1}=1$, then (61) and (60) yield that

63 $$\begin{aligned} {\mathcal{ L}}_{2}:\left( \lambda _{{1}}(j_{{22}}-1)-j_{{21}}\lambda _{{2}} \right) \Phi + \left( 1-j_{{11}} \right) j_{{22}}+j_{{12}} j_{{21}}+j_{{11}}-1=0. \end{aligned}$$

Finally, if ${\mathcal{ R}}_{1}=-1$ and we use (60) and (61), we get

64 $$\begin{aligned} {\mathcal{ L}}_{3}:\left( j_{{21}}\lambda _{{2}}-\lambda _{{1}}(j_{{22}}+1) \right) \Phi + \left( j_{{11}}+1 \right) j_{{22}}-j_{{12}}j_{{21}}+j_{{11}}+1=0. \end{aligned}$$

Hence, the stability region of (57) forms a triangular region bounded by ${\mathcal {L}}_{1}$, ${\mathcal {L}}_{2}$, and ${\mathcal {L}}_{3}$ in the $\lambda _{1}\lambda _{2}$ plane.

## Numerical simulations

In this section, we utilize numerical simulations to validate our theoretical findings and illustrate the complex dynamical behaviors of system (1). We present bifurcation diagrams, phase portraits, and compute the maximum Lyapunov exponents (referred to as ML) to provide a comprehensive understanding of the system’s behavior.

### Example 2

**Figure 3:**
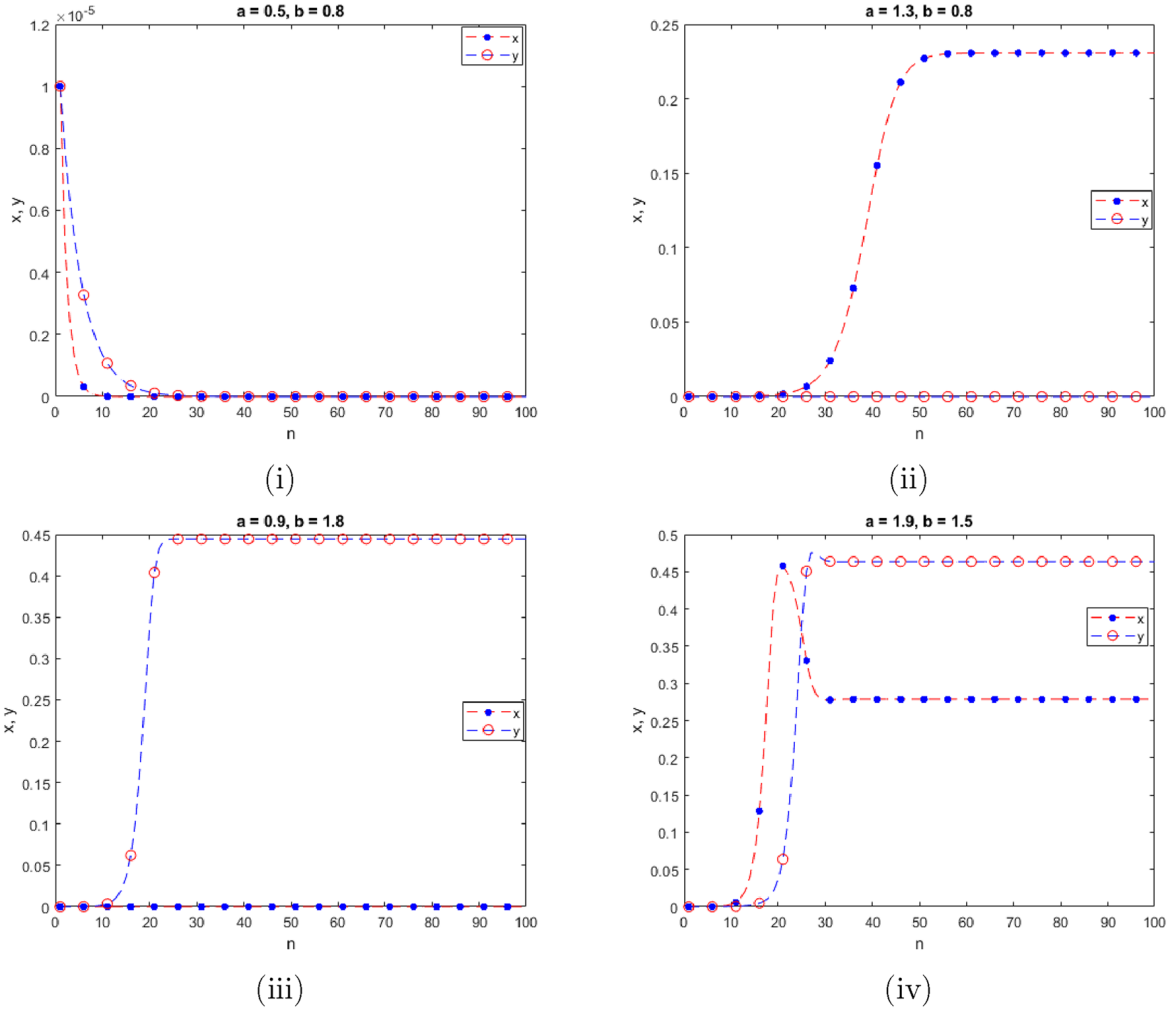
Behavioral characteristics of prey populations $x_{n}$ and predator populations $y_{n}$.

**Figure 4:**
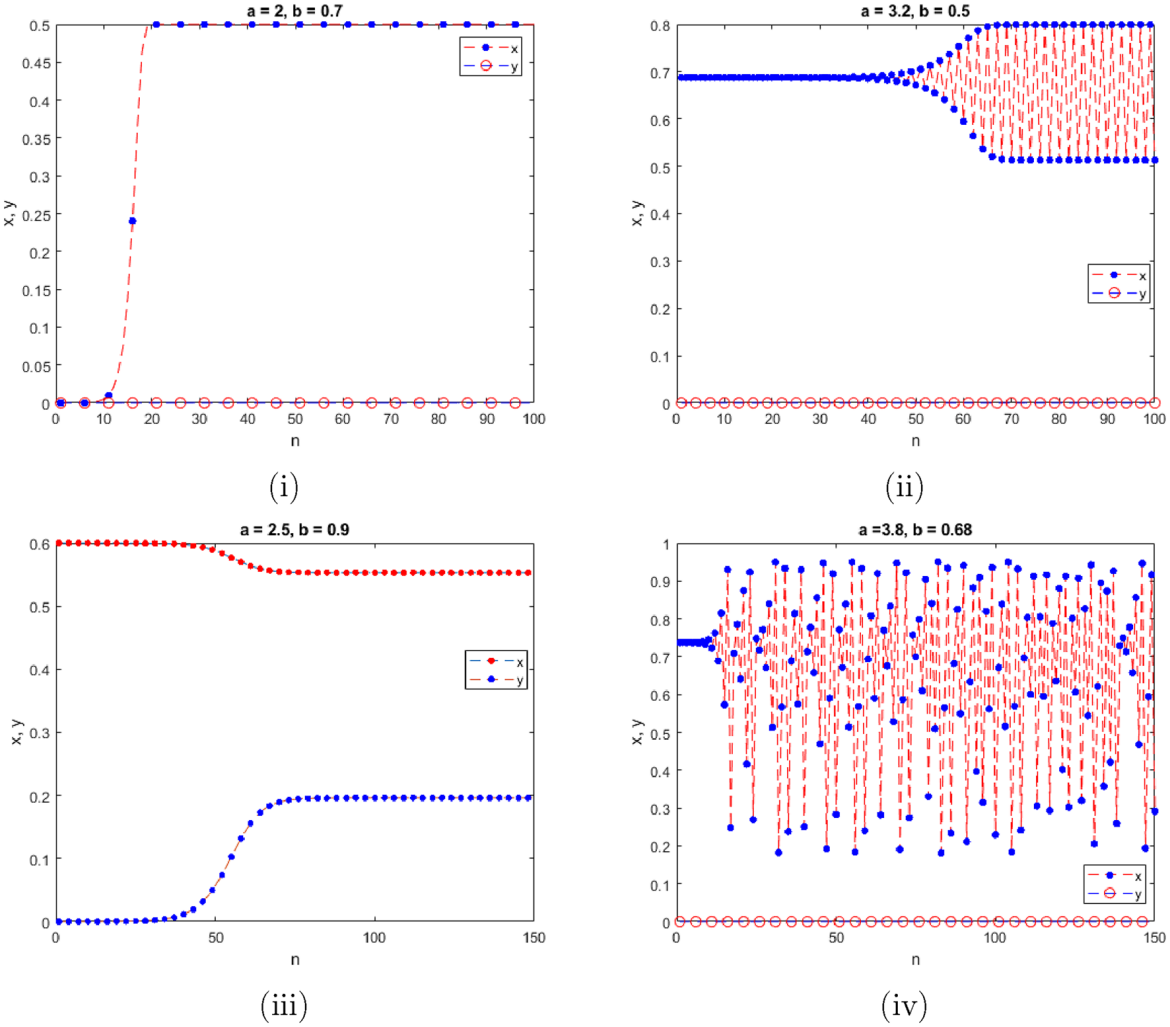
Behavioral characteristics of prey populations $x_{n}$ and predator populations $y_{n}$.

**Figure 5:**
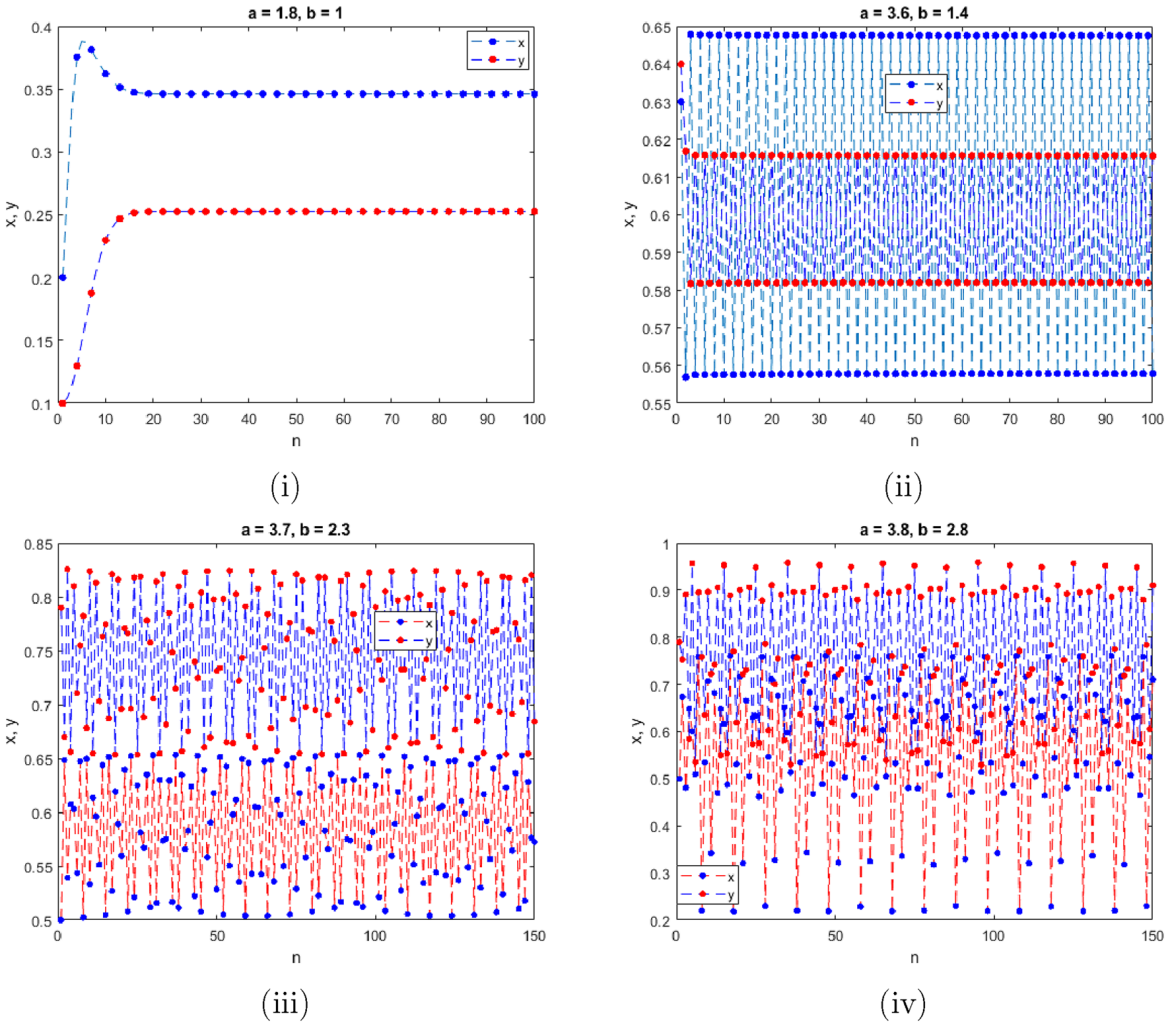
Behavioral characteristics of prey populations $x_{n}$ and predator populations $y_{n}$.

(*I*) Consider parameters a and b while keeping the remaining parameters fixed at c = 0.8, d =0.7. In Fig. 3, we illustrate the relationship between generation n and the populations $x_{n}$ of the prey and $y_{n}$ of the predator to assess their qualitative behavior. In Fig. 3(i), all population curves for the prey $x_{n}$ and the predator $y_{n}$ tend towards zero as n increases, indicating the extinction of both prey and predator when the conditions $0< \hbox {a} = 0.5 < 1$ and $0< \hbox {b} = 0.8 < 1$ are met. This implies that $p_{0}(0.00001, 0.00001)$ is stable. In Fig.3(ii), where $\hbox {a} = 1.3 > 1, \hbox {b} = 0.8 < 1$, the prey’s $x_{n}$ curve goes from zero to infinity, while the prey’s $y_{n}$ curve equals zero. Similarly, in Fig. 3(iii), where $a = 0.9 < 1$, $\hbox {b} = 1.8 > 1$, the prey curve $x_{n}$ equals zero, while the predator’s $y_{n}$ curve goes from zero to infinity, which is unacceptable. Finally, in Fig. 3(iv), where $a = 1.9>1$, $b=1.5> 1$, it’s evident that as the $x_{n}$ curve for prey decreases, the $y_{n}$ curve for the predator increases. This presents a contradiction because the parameters a and b do not satisfy the stability conditions.

(II) Examine parameters a and b while keeping the remaining parameters constant at $c = 0.6$ and $d = 0.5$. In Fig. 4, we illustrate the relationship between generation n and the populations $x_{n}$ of prey and $y_{n}$ of predators to evaluate their specific behavior at $P_{1}(\dfrac{a - 1}{a}, 0)$. In Fig. 4(i), we observe that all population curves for prey $x_{n}$ and predator $y_{n}$ converge to 0.5 and 0, respectively, as n progresses. This indicates the extinction of the predator and the stability of the prey when the conditions $1< \hbox {a} = 2 < 3$ and $0 < \hbox {b} = 0.7<$ $\frac{a+ d - ad}{a}$ are satisfied. This suggests that the $P_{1}(0.5, 0.00001)$ is in a stable state. In Fig. 4(ii), where $\hbox {a} = 3.2 > 3, \hbox {b} = 0.5<$ $\frac{a+ d - ad}{a}$, we observe that both the prey and the predator at (0.6875, 0.00001) with n = 30 are in a stable state. As n increases, we observe that the prey has moved to a periodic state. In this scenario, it becomes unstable, indicating regular prey growth with the predator remaining consistently at zero. In Fig. 4(iii), with $\hbox {a} = 2.5 < 3$ and $\hbox {b} = 0.9>$ $\frac{a+ d - ad}{a}$, it becomes apparent that the xn curve for prey is decreasing, while the yn curve for the predator is increasing. This contradiction arises because the parameters a and b fail to meet the stability conditions. We find that the description of the situation in Fig. 4(iv), where $\hbox {a} = 3.8 > 3, \hbox {b} = 0.68>$ $\frac{a+ d - ad}{a}$, at point (0.7368, 0.00001) is similar to the description in Fig. 3(ii), except that the growth of the prey reaches a stage of chaos and uncontrollability in the absence of the predator.

(III) As in the previous case (*II*), delete the explanation.

(IV) Analyse the parameters a and b, keeping the other parameters constant at c = 0.7 and d = 0.73. In Fig. 5, we illustrate the relationship between generation n and population $x_{n}$ of prey and $y_{n}$ of predators to evaluate their distinct behavior at $p_{3}({\check{x}}, {\check{y}})$. In Fig. 5(i), we observe that, over time, all population curves for prey $x_{n}$ and predator $y_{n}$ converge to 0.346170489 and 0.252704457, respectively. This indicates that both the predator and prey are stable when the conditions a = 1.8 and b = 1 are satisfied, with the initial values (0.2, 0.1) in a steady state. In Fig. 5(ii), where a = 3.6 and b = 1.4, we observe that both the prey and the predator at $p_{3}(0.6875, 0.00001)$ with the initial values (0.5, 0.79) are in an unstable state. We notice that both predator and prey have moved to a cyclic state, which indicates regular growth for both predator and prey. We also employ different values for the parameters a = 3.7, 3.8 and b = 2.3, 2.8 in Fig. 5(iii), (iv), respectively, resulting in curves that do not converge to the initial values (0.5, 0.79). We note two observations. First, when the parameter a (which indicates the growth of the prey) increases by a greater value than the parameter b (which indicates the growth of the predator), this leads to an increase in the number of predators. Second, an increase in the number of prey is matched by an increase in predation, which leads to an increase in the number of prey.

### Example 3

**Figure 6:**
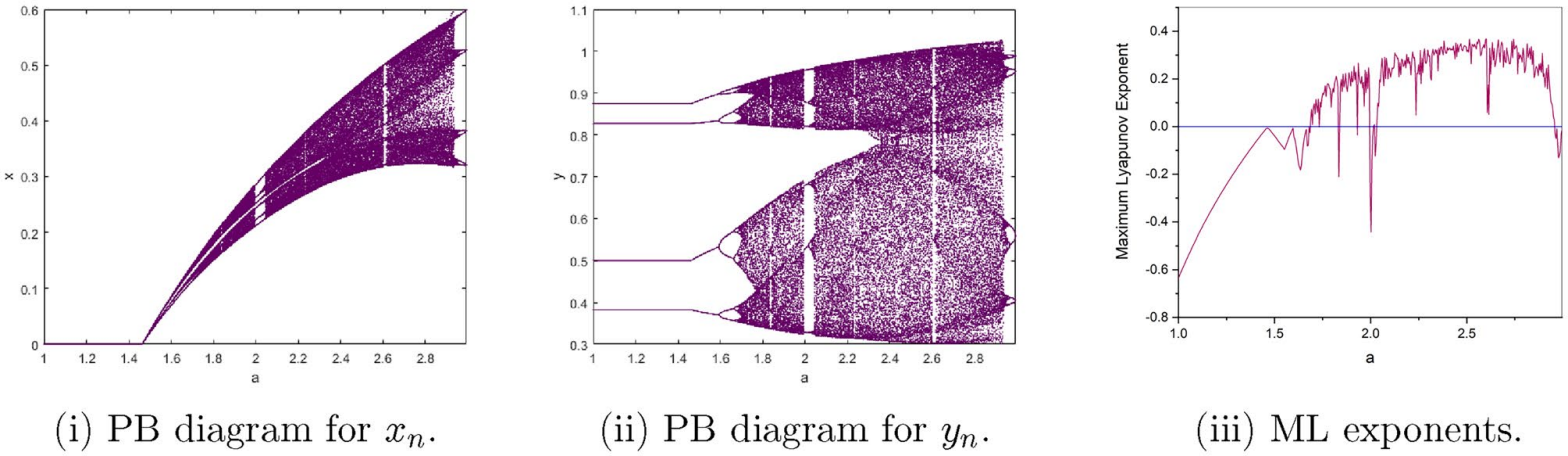
Bifurcation diagram and ML of model (1) for value of $c=0.7, b=3.5, d=0.7, a\in [1, 2.995]$.

**Figure 7:**
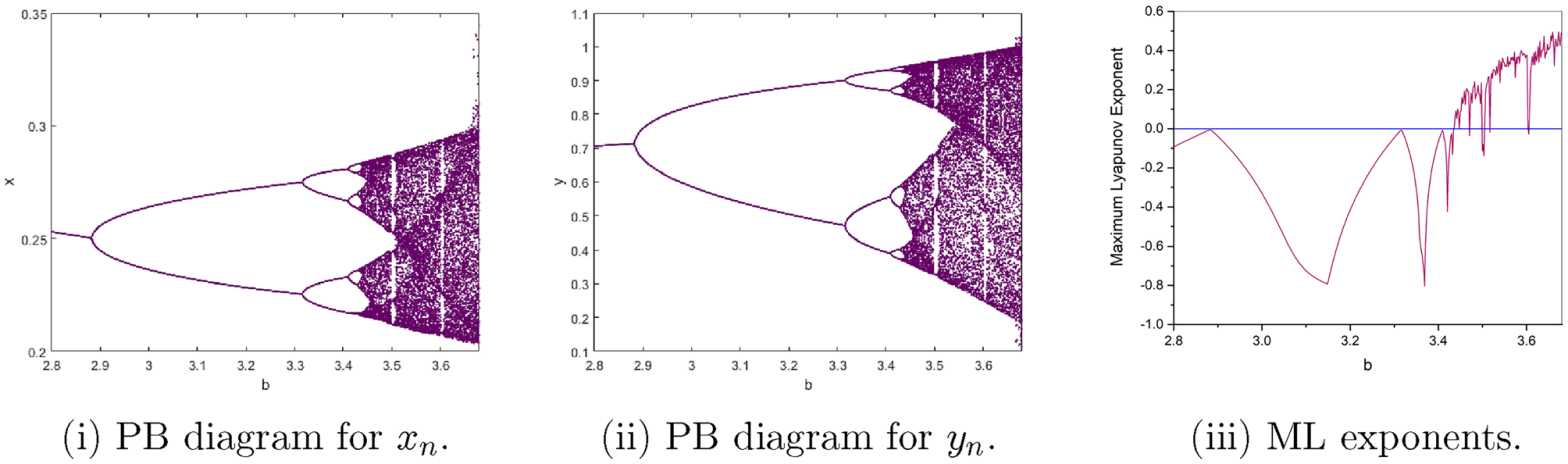
Bifurcation diagram and ML of model (1) for value of $c=0.7, a = 2, d=0.7, b\in [2.8, 3.68]$.

**Figure 8:**
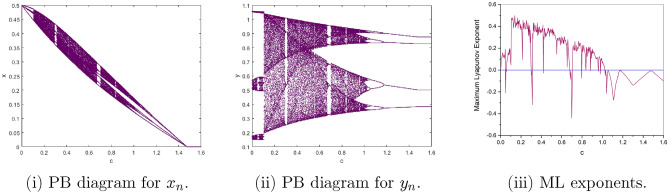
Bifurcation diagram and ML of model (1) for value of $b=3.5, a = 2, d = 0.7, c\in (0, 1.6]$.

**Figure 9:**
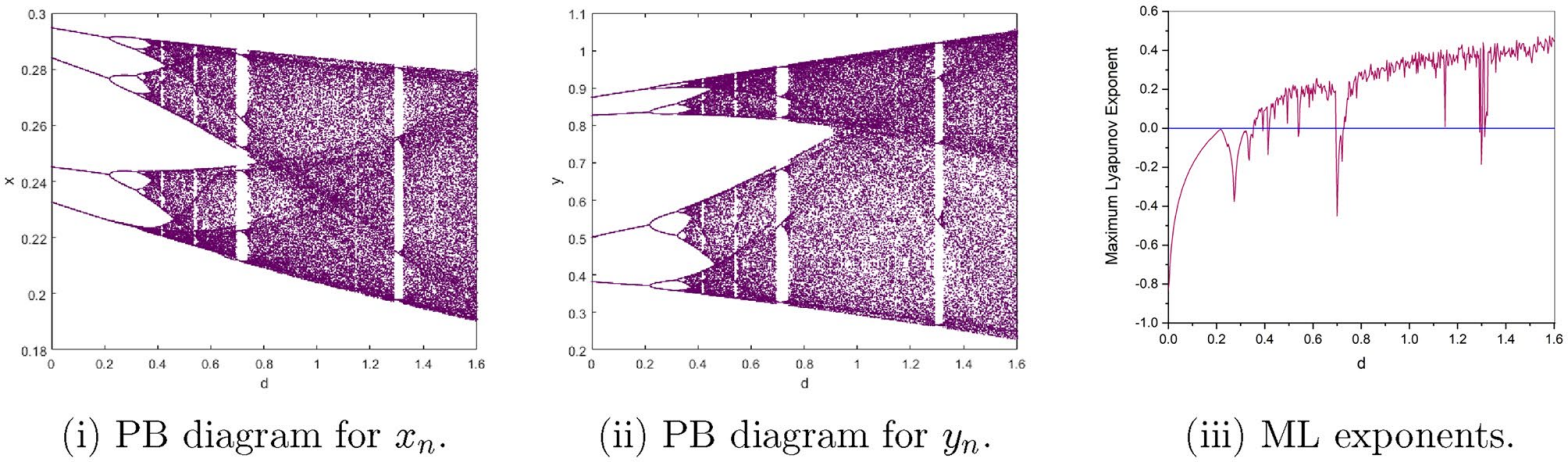
Bifurcation diagram and ML of model (1) for value of $b=3.5, a = 2, c = 0.7, d\in (0, 1.6]$.

(I) At $c=0.7, b=3.5$ and $d=0.7$. Through calculation, the period-doubling bifurcation analysis of model (4) reveals that the $p_{3}({\check{x}}, {\check{y}})$ occurs at a = 2, with $\Psi _{2}= -0.4066927936$ and $\Psi _{1}= 29.66966761$, while the parameters $(a, b, d, c) \in F_{P_{3}}$.We observe from Fig. 6(i), (ii) that the fixed point $p_{3}({\check{x}}, {\check{y}})$ remains stable for $1 \le a < 1.46$ and loses its stability at the period-doubling bifurcation parameter value a = 1.46. Furthermore, we observe the presence of periodic orbits with periods of 4, 6, 7, 8, and 16. The corresponding maximum Lyapunov exponents for Fig. 6(i), (ii) are depicted in Fig. 6(iii).

(II) When $c = 0.7, a = 2$ and $d = 0.7$ are set, we observe from Fig. 7(i), (ii) that the fixed point $p_{3}({\check{x}}, {\check{y}})$ remains stable within the range $2.8 \le b < 2.881$ and loses stability at b = 2.881, representing the period-doubling bifurcation. Additionally, orbits with periods of 2, 4, 5, 6, 8, 10, and 16 are present. Figure 7(iii) illustrates the corresponding maximum Lyapunov exponents from Fig. 7(i), (ii).

(III) When taking the parameters $b = 3.5, a = 2$, and $d = 0.7$, we observe from Fig. 8(i), (ii) that the fixed point $p_{3}({\check{x}}, {\check{y}})$ remains stable within the range $1.455 < c \le 1.6$ and loses its stability at the value c = 1.455, representing the period-doubling bifurcation. Moreover, orbits with periods of 4, 5, 6, 7, 8, 10 and 16 are also present. Fig. 8(iii) illustrates the corresponding maximum Lyapunov exponents from Fig. 8(i), (ii).

(IV) For the parameter values $c=0.7$, $b=3.5$, and $a=2$, we observe from Fig. 9(i), (ii) that the fixed point $p_{3}({\check{x}}, {\check{y}})$ remains stable for $0< d < 0.216$, but loses its stability at the bifurcation parameter value $d = 0.216$, indicating a period-doubling bifurcation. Moreover, we observe the presence of periodic orbits with periods of 5, 6, 7, 8, 10, 12, and 16. The corresponding maximum Lyapunov exponents for Fig. 9(i), (ii) are illustrated in Fig. 9(iii).

### Example 4

**Figure 10:**
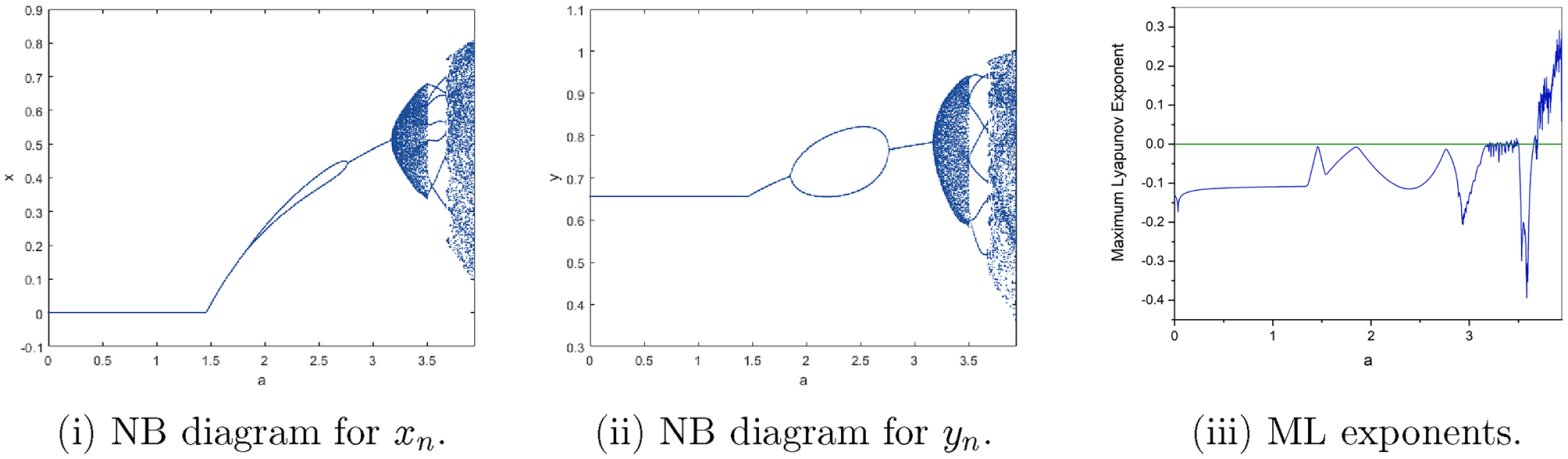
Bifurcation diagram and ML of model (1) for value of $c=0.7, b=2.9, d=0.73, a\in (0, 3.9425]$.

**Figure 11:**
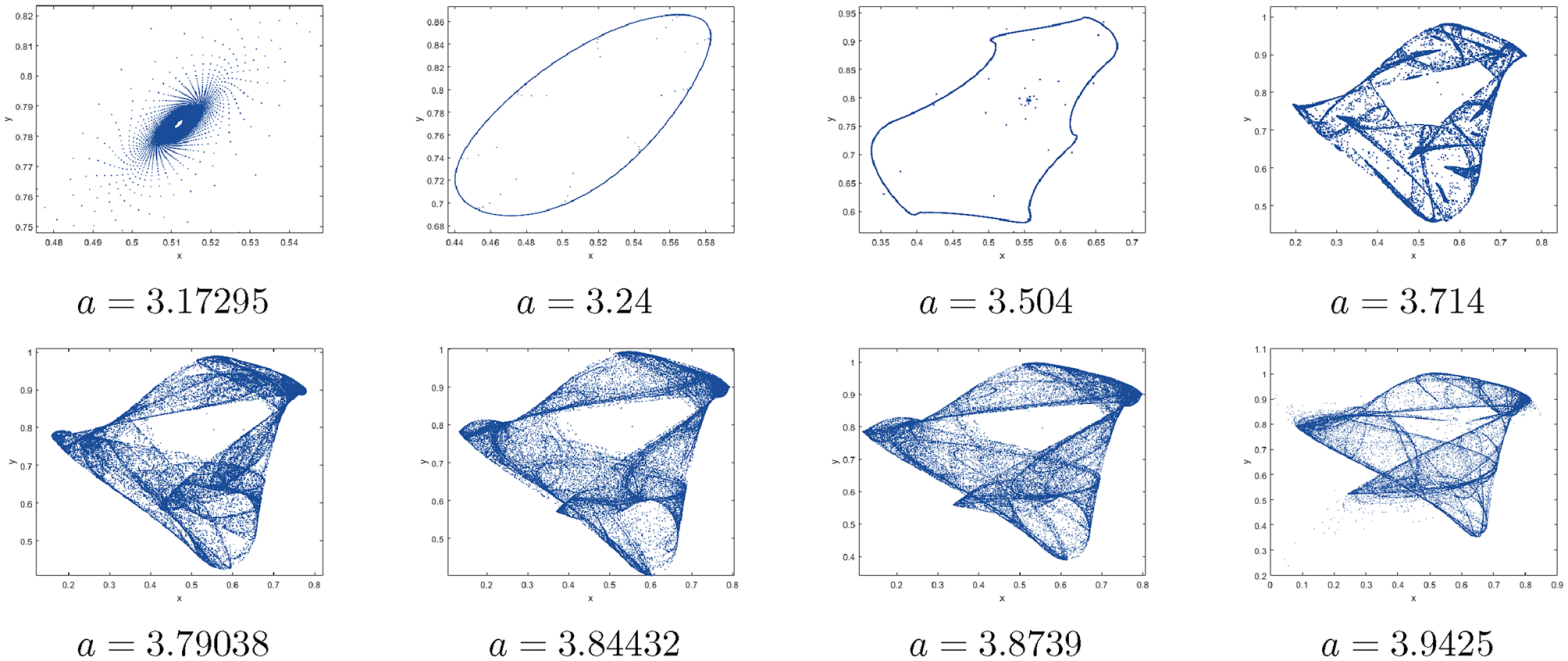
The phase pictures associated with Fig. 10(i), (ii).

**Figure 12:**
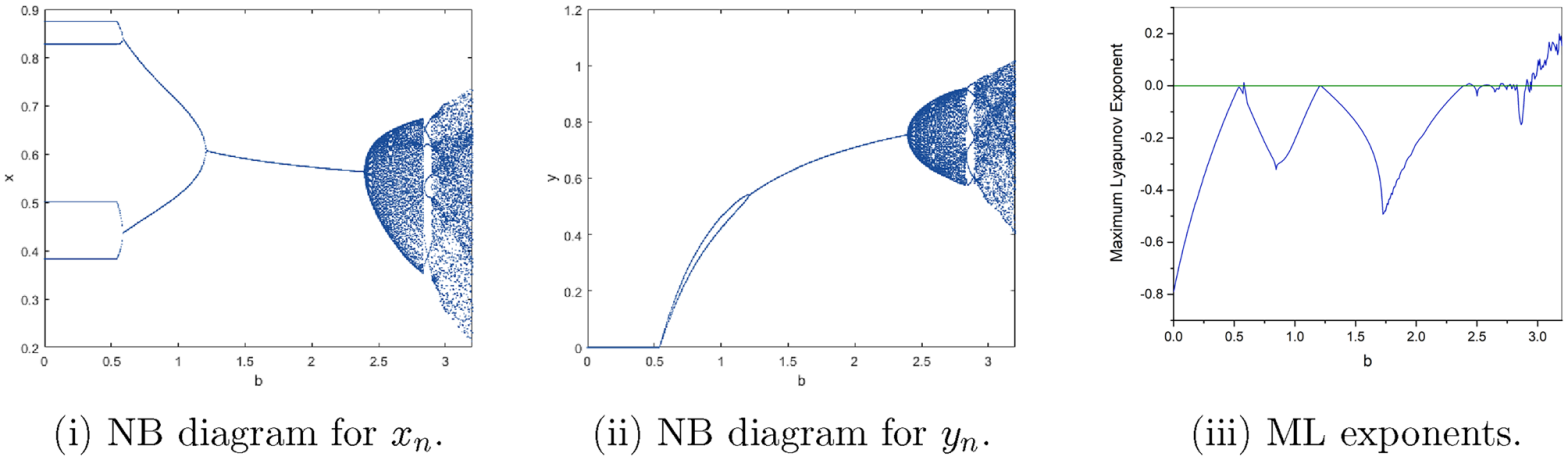
Bifurcation diagram and ML of model (1) for value of $c=0.7, a = 3.5, d = 0.73, b\in (0, 3.2]$.

**Figure 13:**
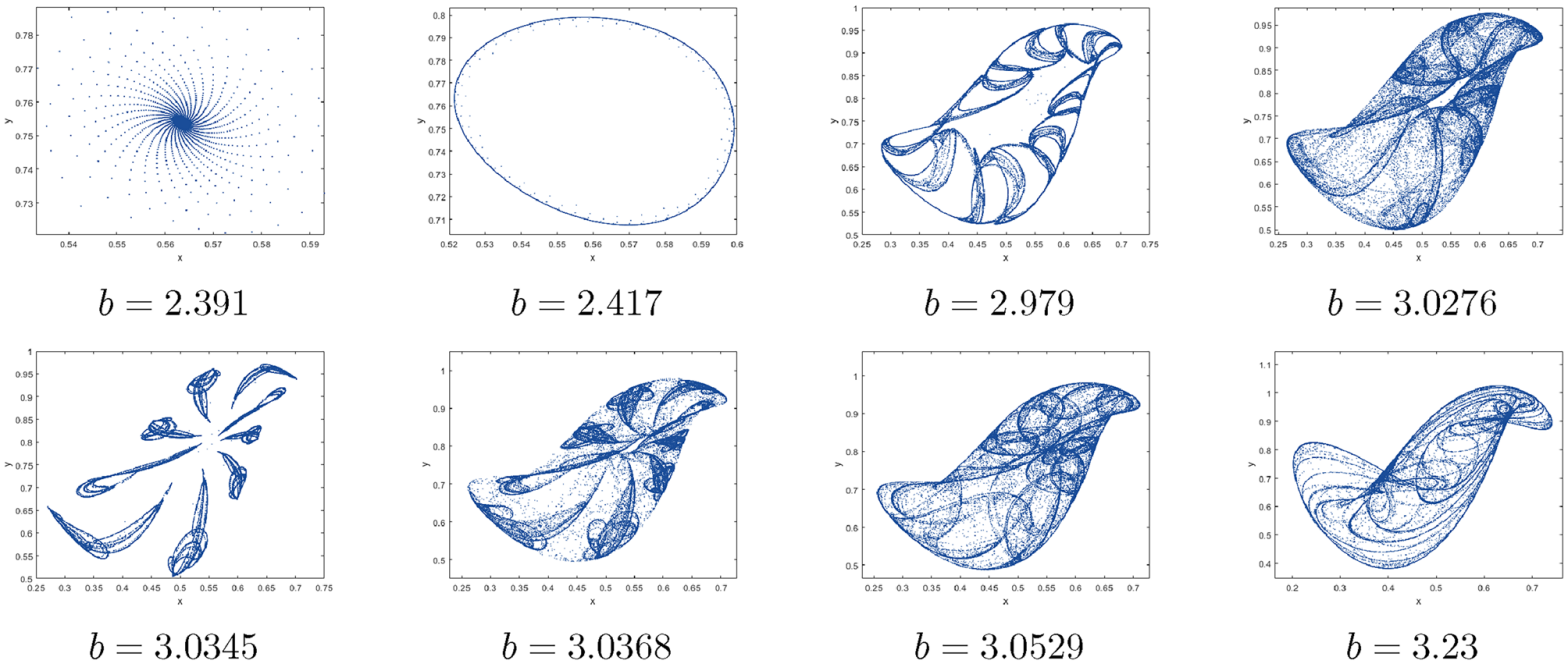
The phase pictures associated with Fig. 12(i), (ii).

**Figure 14:**
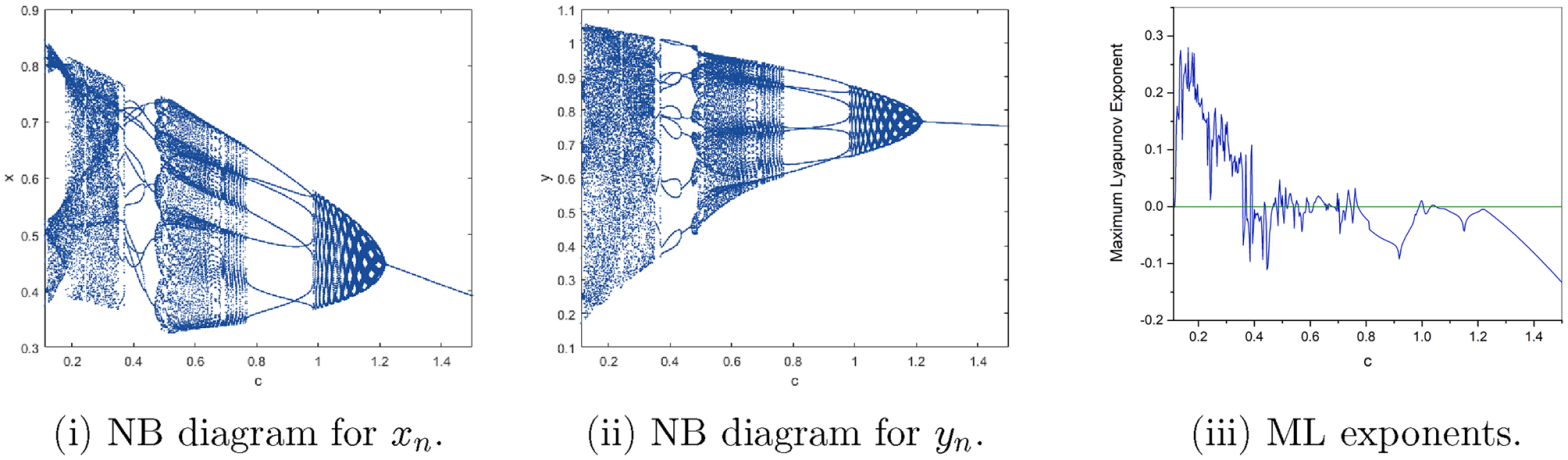
Bifurcation diagram and ML of model (1) for value of $b=2.9, a =3.5, d = 0.73, c\in [0.112, 1.5]$.

**Figure 15:**
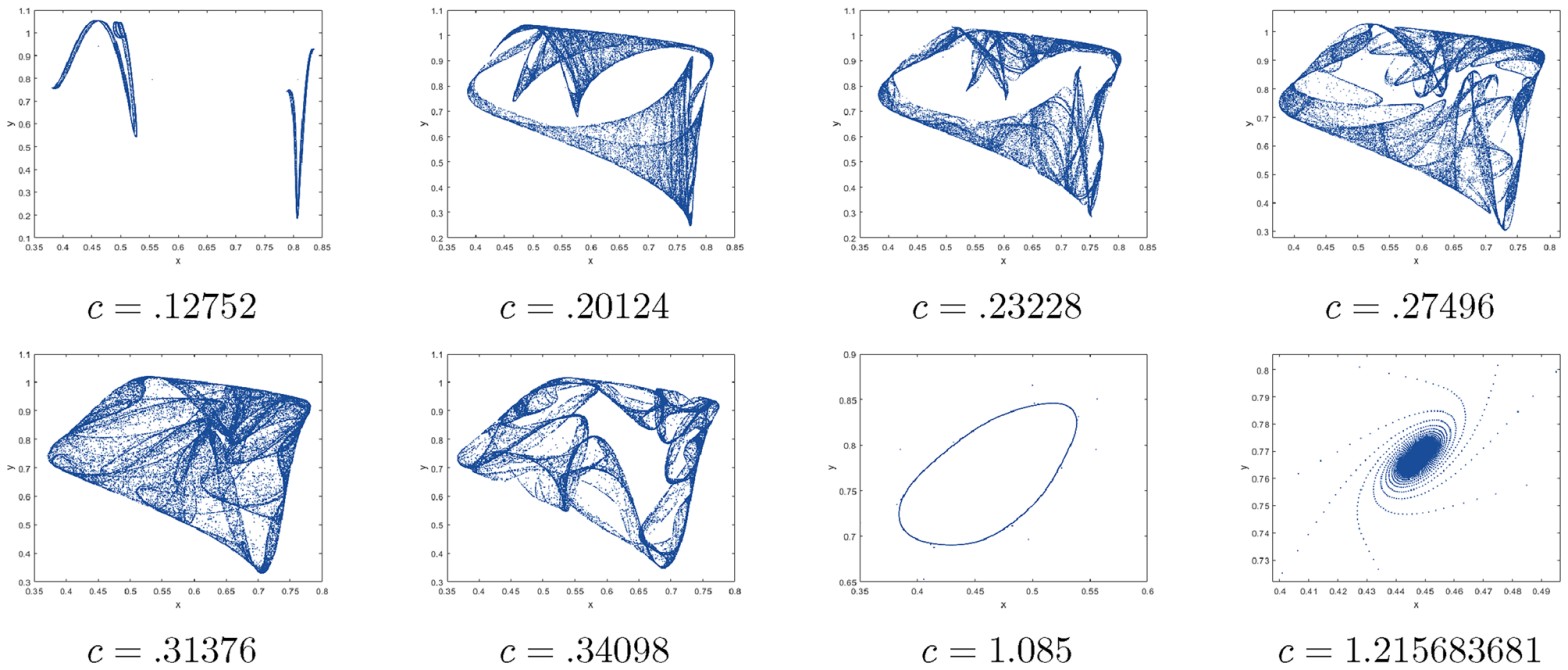
The phase pictures associated with Fig. 14(i), (ii).

**Figure 16:**
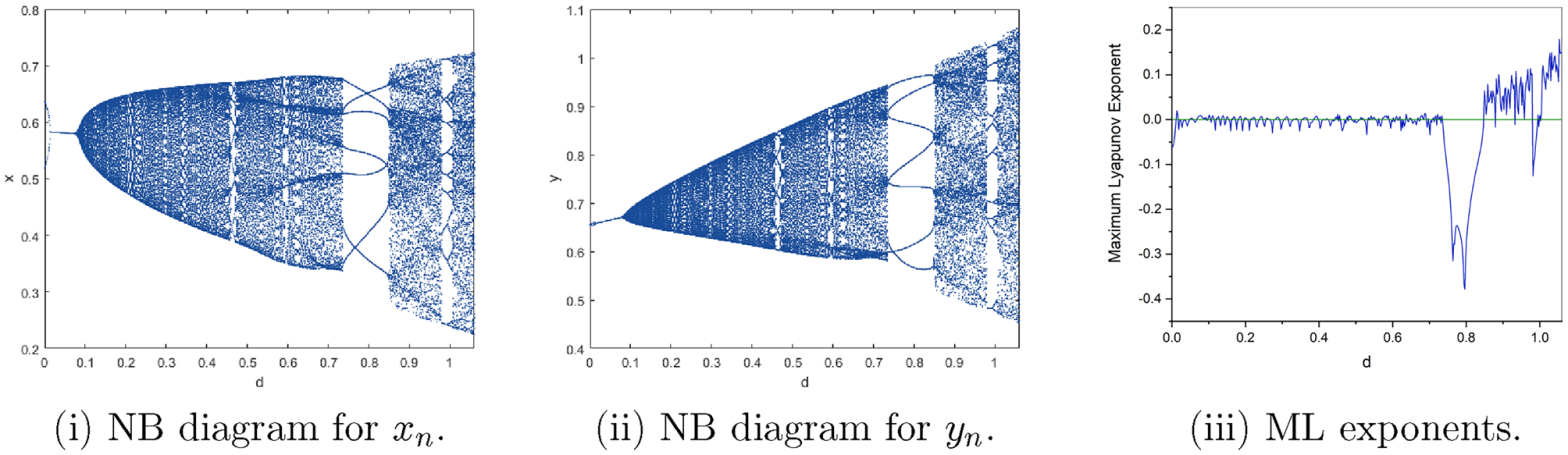
Bifurcation diagram and ML of model (1) for value of $b=2.9, a = 3.5, c = 0.7, d\in (0, 1.06]$.

**Figure 17:**
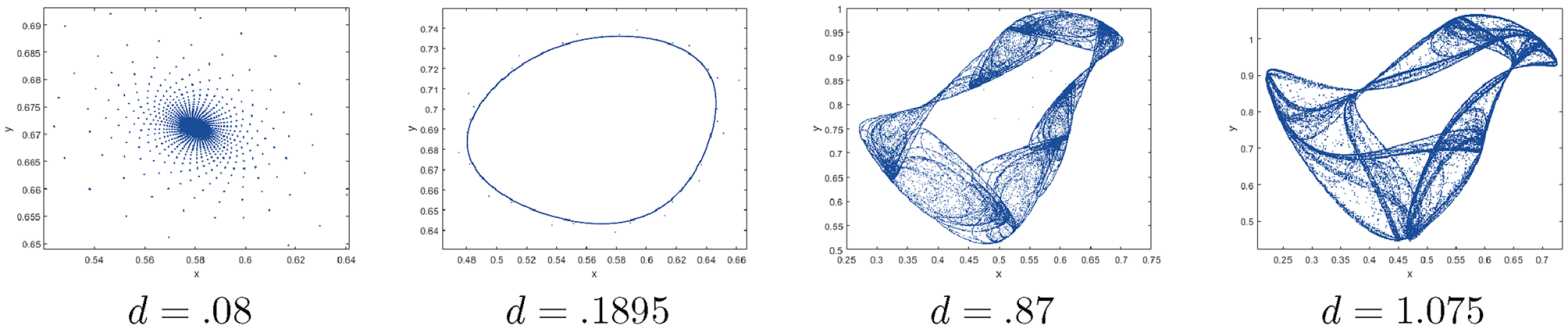
The phase pictures associated with Fig. 16(i), (ii).

(I) For $c=0.7, b=2.9$ and $d=0.73$. Through calculation, the Neimark-Sacker bifurcation analysis of model (4) reveals that the $p_{3}({\check{x}}, {\check{y}})$ occurs at $a = 3.5$, with ${\mathfrak{f}}$ = −1.838901649, while the parameters $(a, b, d, c) \in N_{P_{3}}$. We observe from Fig. 10(i), (ii) that the fixed point $p_{3}({\check{x}}, {\check{y}})$ is stable within the parameter range of $0< a < 3.174$. However, the fixed point loses stability at the Neimark–Sacker bifurcation parameter value $a = 3.174$, as shown in Fig. 10(i), (ii). The maximum Lyapunov exponents, represented in Fig. 10(iii), correspond to the dynamics depicted in Fig. 10(i), (ii). Furthermore, Fig. 11 displays the phase portraits corresponding to Fig. 10(i), (ii).

(II) At $c = 0.7, a = 3.5$ and $d = 0.73$ are set, we observe from Fig. 12(i), (ii) that the fixed point $p_{3}({\check{x}}, {\check{y}})$ remains stable within the range $0< b < 2.4$. However, the fixed point loses stability at the Neimark–Sacker bifurcation parameter value $b = 2.4$, as illustrated in Fig. 12(i), (ii). The maximum Lyapunov exponents corresponding to Fig. 12(i), (ii) are shown in Fig. 12(iii). Additionally, Fig. 13 displays the phase portraits corresponding to Fig. 12(i), (ii).

(III) When $b = 2.9, a=3.5$ and $d = 0.73$, we observe stability of the fixed point $p_{3}({\check{x}}, {\check{y}})$ in the parameter range of $1.216 < c \le 1.5$, but it loses stability at the Neimark–Sacker bifurcation parameter value c = 1.216. The maximum Lyapunov exponents, depicted in Fig. 14(iii), are related to the dynamics shown in Fig. 14(i), (ii). Additionally, Fig. 15 presents the phase portraits corresponding to Fig. 14(i), (ii).

(IV) If $b = 2.9, a=3.5$ and $c = 0.7$, we observe that the fixed point $p_{3}({\check{x}}, {\check{y}})$ remains stable within the parameter range of $0< d < 0.85$. However, at the Neimark–Sacker bifurcation parameter value $d = 0.85$, the system undergoes instability. The maximum Lyapunov exponents corresponding to Fig. 16(i), (ii) are depicted in Fig. 16(iii). Moreover, Fig. 17 presents the phase portraits corresponding to Fig. 16(i), (ii).

### Chaos control

In the subsection on chaos control for model (1), we employ the OGY method and choose the parameters $a = 3.5$, $b = 2.9$, $c = 0.7$, and $d = 0.73$. As a result, model (1) exhibits an unstable fixed point at (0.5552950005, 0.7949535691). Taking $c_{0}=0.7$ as the nominal value, the controlled model can be expressed as follows:

65 $$\begin{aligned} \begin{array}{l} x_{n+1}=ax_{n}(1-x_{n})-(c\lambda _{1}(x_{n}-{\check{x}})-\lambda _{2}(y_{n}-{\check{y}}))x_{n}y_{n}, \\ y_{n+1}=by_{n}(1-y_{n})+dx_{n}y_{n}, \end{array} \end{aligned}$$

where $$\mathcal \lambda =\left[ \begin{array}{cc} \lambda _{1}&\lambda _{2} \end{array} \right]$$ and (0.5552950005, 0.7949535691) is unstable fixed point of model (1 ). Furthermore, we have

$$\begin{aligned} J=\left[ \begin{array}{ccc} -0.9435325016 &{} &{} -0.3887065003 \\ &{} &{} \\ 0.5803161054 &{} &{} -1.305365350 \end{array} \right] , \end{aligned}$$

$$\begin{aligned} {\mathcal{ L}}=\left[ \begin{array}{c} -0.4414337425 \\ \\ 0 \end{array} \right] , \end{aligned}$$

and

$$\begin{aligned} \tau =\left[ {\mathcal{ L}}:J{\mathcal{ L}}\right] =\left[ \begin{array}{ccc} -0.4414337425 &{} &{}0.4165070833 \\ &{} &{} \\ 0 &{} &{} -0.2561711102 \end{array} \right] . \end{aligned}$$

The rank of $\tau$ can be readily observed to be 2, indicating that the model (65) is controllable. Additionally, the Jacobian matrix is given by:

66 $$\begin{aligned} J-{\mathcal{ L}}\lambda =\left[ \begin{array}{cc} -0.9435325016 + 0.4414337425\lambda _{1} &{}-0.3887065003 + 0.4414337425\lambda _{2}\\ &{} \\ 0.5803161054 &{}-1.305365350 \end{array} \right] . \end{aligned}$$

Hence, the characteristic equation of (66) can be expressed as follows:

67 $$\begin{aligned} \varvec{\lambda }({\mathcal{ R}})= & {} {\mathcal{ R}}^{2}-(0.4414337425\lambda _{1}-2.248897852){\mathcal{ R}} \nonumber \\{} & {} +1.457227276 - 0.5762323118\lambda _{1}-0.2561711102\lambda _{2}. \end{aligned}$$

Thus, the lines of marginal stability are given as follows:

$$\begin{aligned} \begin{array}{l} {\mathcal{ L}}_{1}:0.457227277=0.5762323119\lambda _{1}+0.2561711102\lambda _{2},\\ \\ {\mathcal{ L}}_{2}:4.706125129=1.017666054\lambda _{1}+0.2561711102\lambda _{2}, \end{array} \end{aligned}$$

and

$$\begin{aligned} \begin{array}{l} {\mathcal{ L}}_{3}:0.208329425=0.1347985694\lambda _{1}+0.2561711102\lambda _{2}. \end{array} \end{aligned}$$

**Figure 18:**
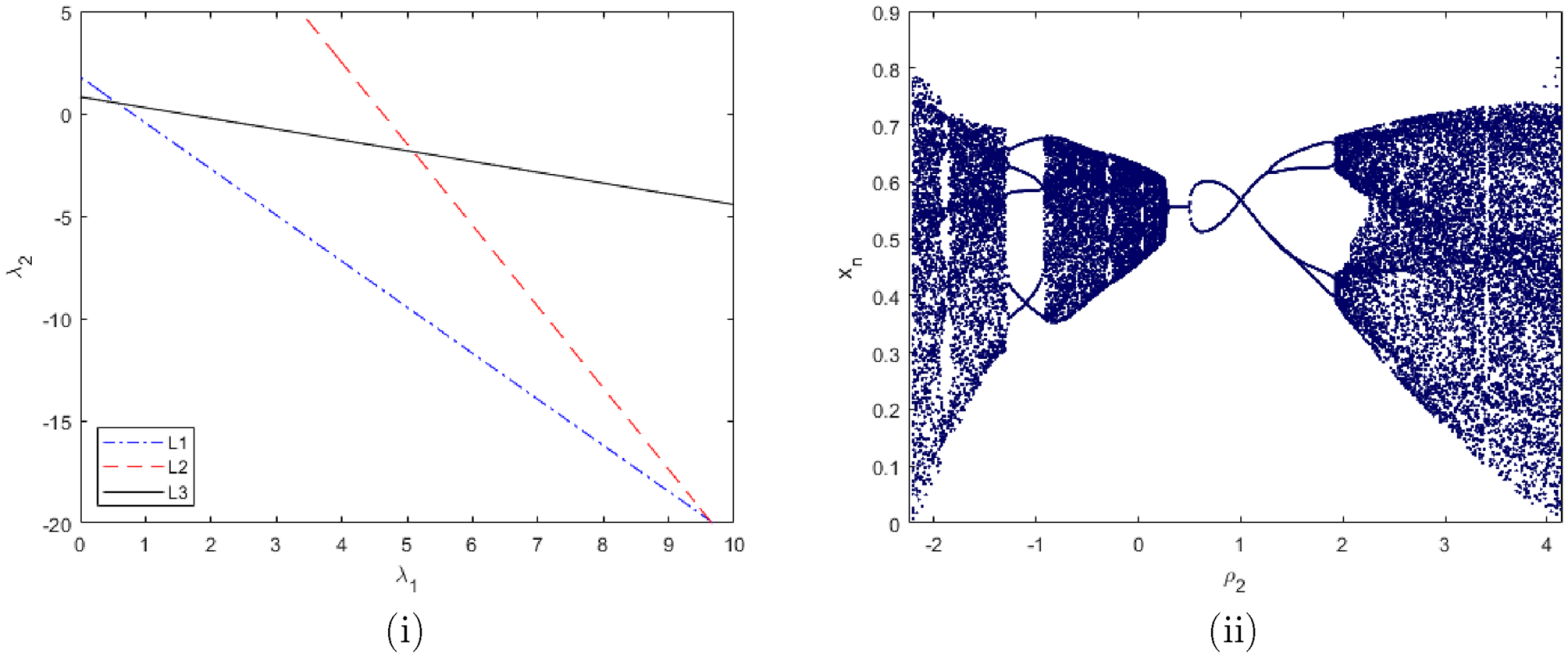
(i) Triangular stability region bounded by ${\mathcal{ L}}_{1},{\mathcal{ L}}_{2}$ and ${\mathcal{ L}}_{3}$ of the controlled system (65). (ii) Bifurcation diagrams for the controlled system (65) with $\lambda _{1}=0.6\;and\; \lambda _{2}\in [-2.25, 4.15]$.

Figure 18(i) illustrates the stable triangular region formed by the marginal lines ${\mathcal {L}}_{1}$, ${\mathcal {L}}_{2}$, and ${\mathcal {L}}_{3}$ for the controlled model (65).

Choosing $\lambda _{1}=0.6$ and $\lambda _{2}\in [-2.25, 4.15]$. The controlled model’s bifurcation diagrams (65) are then illustrated in Fig. 18(ii).

## Conclusion

This model represents predator-prey interactions that play a vital role in maintaining stability and biodiversity in an ecosystem. An increased population density of prey leads to instability in the ecosystem in the absence of predators. The opposite is true: in the absence of prey, this leads to the extinction of predators and, thus, the collapse of the ecosystem. Predation is a directly proportional relationship between predator and prey; when prey is abundant, predators increase, and thus the number of prey through predation decreases. Conversely, when predators are abundant, prey is scarce, and thus the number of predators decreases through starvation, intensifying the struggle for prey. (Mating between males is usually an aggressive act towards females that sometimes results in serious injury or death, but in those species in which several females become fertile at the same time, there can be a strong element of competition and dominance for access to females for reproduction). In this study, we employed coupled logistic maps and difference equations to qualitatively analyse a discrete-time prey-predator model. Our main objective was to explore the model’s behaviour by investigating various parameter values and initial conditions. We focused on the emergence of stable equilibria, period doubling, and chaotic attractors, as well as the analysis of codimension-one bifurcations, including Marotto’s chaos and chaos control. Our analysis revealed a wide range of captivating dynamic behaviours, highlighting the model’s sensitivity to variations in key parameters. These findings have significant implications for our understanding of prey-predator systems. The bifurcation phenomena in such systems can be studied using the center manifold theorem. In this paper, we took a step towards understanding the dynamics of these systems by investigating local and global bifurcations in a two-dimensional normal form map derived from the proposed model. We conducted a detailed investigation of the nature of eigenvalues and calculated period-doubling and Neimark-Sacker bifurcations. We also derived conditions for the occurrence of local codimension-one bifurcations. To demonstrate the occurrence of various bifurcations in this map, we presented numerical examples. Overall, our study contributes to the understanding of the dynamic behaviours exhibited by prey-predator models and provides insights into the occurrence of different bifurcations in such systems. By uncovering the intricate dynamics of these systems, the research contributes to our broader knowledge of ecological systems and their behaviour.

In the future, a deeper understanding of ecosystem stability can be attained through the exploration of the global dynamics of the predator-prey model. By systematically varying parameters and employing codimension-2 bifurcation analysis, we can uncover novel bifurcation phenomena and their implications for population dynamics. Additionally, in light of the substantial influence of the Allee effect on predator populations, future research endeavors can prioritize its investigation.

## Data Availability

All the data used in this manuscript has been presented within the article.

## Code availability

The codes used and/or analysed during the current study are available from the corresponding author on reasonable request.
